# Molecular evidence of hybridization in sympatric populations of the *Enantia jethys* complex (Lepidoptera: Pieridae)

**DOI:** 10.1371/journal.pone.0197116

**Published:** 2018-05-17

**Authors:** Jovana M. Jasso-Martínez, Salima Machkour-M’Rabet, Roger Vila, Rosario Rodríguez-Arnaiz, América Nitxin Castañeda-Sortibrán

**Affiliations:** 1 Laboratorio de Genética y Evolución, Departamento de Biología Celular, Universidad Nacional Autónoma de México, Ciudad de México, Mexico; 2 Laboratorio de Ecología Molecular y Conservación, Departamento de Conservación de la Biodiversidad, El Colegio de la Frontera Sur, Chetumal, Quintana Roo, Mexico; 3 Institut de Biologia Evolutiva (CSIC-UPF), Barcelona, Spain; Leibniz-Institute of Freshwater Ecology and Inland Fisheries, GERMANY

## Abstract

Hybridization events are frequently demonstrated in natural butterfly populations. One interesting butterfly complex species is the *Enantia jethys* complex that has been studied for over a century; many debates exist regarding the species composition of this complex. Currently, three species that live sympatrically in the Gulf slope of Mexico (*Enantia jethys*, *E*. *mazai*, and *E*. *albania*) are recognized in this complex (based on morphological and molecular studies). Where these species live in sympatry, some cases of interspecific mating have been observed, suggesting hybridization events. Considering this, we employed a multilocus approach (analyses of mitochondrial and nuclear sequences: *COI*, *RpS5*, and *Wg;* and nuclear dominant markers: inter-simple sequence repeat (ISSRs) to study hybridization in sympatric populations from Veracruz, Mexico. Genetic diversity parameters were determined for all molecular markers, and species identification was assessed by different methods such as analyses of molecular variance (AMOVA), clustering, principal coordinate analysis (PC_o_A), gene flow, and *PhiPT* parameters. ISSR molecular markers were used for a more profound study of hybridization process. Although species of the *Enantia jethys* complex have a low dispersal capacity, we observed high genetic diversity, probably reflecting a high density of individuals locally. ISSR markers provided evidence of a contemporary hybridization process, detecting a high number of hybrids (from 17% to 53%) with significant differences in genetic diversity. Furthermore, a directional pattern of hybridization was observed from *E*. *albania* to other species. Phylogenetic study through DNA sequencing confirmed the existence of three clades corresponding to the three species previously recognized by morphological and molecular studies. This study underlines the importance of assessing hybridization in evolutionary studies, by tracing the lineage separation process that leads to the origin of new species. Our research demonstrates that hybridization processes have a high occurrence in natural populations.

## Introduction

At the beginning of 20th century, hybrid specimens were considered rare in nature and a phenomenon with little evolutionary importance [1]. Federley [2] pointed out that hybrids between the butterflies *Pygaera pigra* and *P*. *curtula* were chromosomally incompatible and short-lived. Current studies have shown that hybridization is not a rare process in nature and is evidence of the evolutionary speciation process, wherein population splitting lead to the subsequent divergence of entire lineages [3]. In animal species, hybridization is common, with hybridization reported in at least 10% of all animal species, predominantly in the more recent species [4]. Hybridization can be defined as the production of viable hybrids from interspecific mating, and introgression as the integration of foreign genetic material from one species into another through backcrossing [5]. Hybridization events have been repeatedly demonstrated in natural butterfly populations. For example, Lushai and colleagues [6–7] revealed the existence of a hybrid zone between the tropical *Danaus chrysippus* (L.) subspecies. They suggested that hybridism was reinforced by the action of a bacterial symbiont male-killer, *Spiroplasma*, which forced females in female-biased populations to mate with heterotypic males. Furthermore, these studies suggest that hybridization events could result in polyphyletic species. Similarly, Mullen and colleagues [8] demonstrated the existence of a hybrid zone between mimetic and non-mimetic populations of the polytypic *Limenitis arthemis-astyanax* species complex using mitochondrial and nuclear sequences. Other studies have demonstrated hybridization and introgression events among Neotropical *Heliconius* species using mitochondrial and nuclear sequences [9], DNA sequences and amplified fragment length polymorphism (AFLP) [10], and genome-wide genotypic and DNA sequences [11], among others. These studies have shown that interspecific gene flow may remain high even after speciation, and that these events (hybridization and introgression) are important factors for the evolution of animal species, as well as a source of genetic variability. Therefore, a new view of hybridization has emerged and is currently regarded as not only forces that oppose diversification and species stability, but to also have the potential to increase biodiversity [12–13].

*Enantia* is a genus of butterflies belonging to the Pieridae family and composed of nine species with a Neotropical distribution [14]. In Mexico, Llorente-Bousquets [15] defined the *Enantia jethys* complex to be composed of three species with a sympatric distribution that encompasses the Mexican Gulf slopes of the Sierra Madre Occidental mountain range: *Enantia jethys* Boisduval, 1836; *Enantia mazai* Llorente-Bousquets, 1984; and *Enantia albania* Bates, 1864. *Enantia mazai* and *E*. *jethys* are endemic to Mexico [14–15]. The principal characteristics defining this species complex are genitalia morphology and wing pattern pigmentation [15]. Specifically, male individuals of each species in the *Enantia jethys* complex present characteristic wing pigmentation patterns with seasonal variation (i.e., in the wet season they have tonalities closer to orange, while in the dry season, tonalities are closer to yellow) [15]. In addition, to distinguish between female *E*. *jethys* and *E*. *mazai*, a detailed examination of the wing pigmentation patterns is required. Besides occurring in sympatry, all species of the complex share the same habitat (principally cloud forests), and they oviposit on the same host plants (*Inga* spp.) [15].

For over a century, there have been numerous debates on the identification of the species belonging to the *Enantia jethys* complex (see [15] for a review). More recently, molecular studies have contributed to the understanding of the relationship among the different species in Mexico. Castañeda-Sortibrán [16], using allozymes, confirmed the existence of three distinct species (*E*. *albania*, *E*. *jethys*, and *E*. *mazai*) in the sympatric area of the Mexican Gulf slope, with signals of gene flow among species. Using the mitochondrial cytochrome c oxidase subunit I (*COI*), Jasso-Martínez and colleagues [17] identified some specimens morphologically indistinguishable from *E*. *albania* (cryptic species), but forming a sister clade of *E*. *mazai*, suggesting the possibility of recent speciation or even hybridization. The existence of hybridization is supported by field expeditions that have permitted the observation of two separate events of interspecific mating: one between male *E*. *jethys* and female *E*. *mazai*, and another between male *E*. *albania* and female *E*. *jethys* (personal observation, Castañeda-Sortibrán and Jasso-Martínez in this paper), suggesting that hybridization may be a relatively frequent process in the *Enantia jethys* complex.

In this study, we employed the molecular markers commonly used in phylogenetic studies (mitochondrial and nuclear sequences), as well as inter-simple sequence repeat (ISSR) markers to examine contemporary genetic exchange among the butterflies of the *Enantia jethys* complex. Identification of fast molecular markers, such as ISSR, are the principal tools to study hybridization processes, and are recommended instead of using microsatellites [18]. Hybridization and introgression events have been successfully studied through ISSRs in many taxonomic groups [19–22], including butterflies [23]. The ISSR-PCR method is a molecular technique used to screen a large part of the genome without prior knowledge of the sequences. This method provides highly reproducible results and generates abundant polymorphisms in many systems [20]. The ISSRs are dominant molecular markers, where the absence of a band is interpreted as the loss of a locus/allele through either the deletion of the SSR site or a chromosomal rearrangement [24]. The use of ISSR to study species of Lepidoptera is relatively recent, yet abundant (not an exhaustive list: [25–31]).

In summary, considering the hypothesis of hybridization events in the *Enantia jethys* complex, we used a set of molecular markers (one mitochondrial and two nuclear sequences, and ISSR) to: i) confirm the hypothesis of the hybridization process in the butterfly complex, and, in the event our hypothesis is correct, to evaluate the extent of this process; ii) identify the directionality of the introgression; iii) determine if genetic diversity presents variation between admixed and non-admixed individuals (i.e., hybrids and non-hybrids) for each morphospecies; and iv) evaluate, using different molecular markers, the monophyly state of the butterfly complex. Finally, we discuss different hypotheses to explain the evolutionary history of this complex.

## Materials and methods

### Ethics statement

Species involved in this study are not endangered or protected in the study area, and no specific permission is required for scientific research. No specific permission was required for our fieldwork in any location visited.

### Butterfly samples

**Fig 1:**
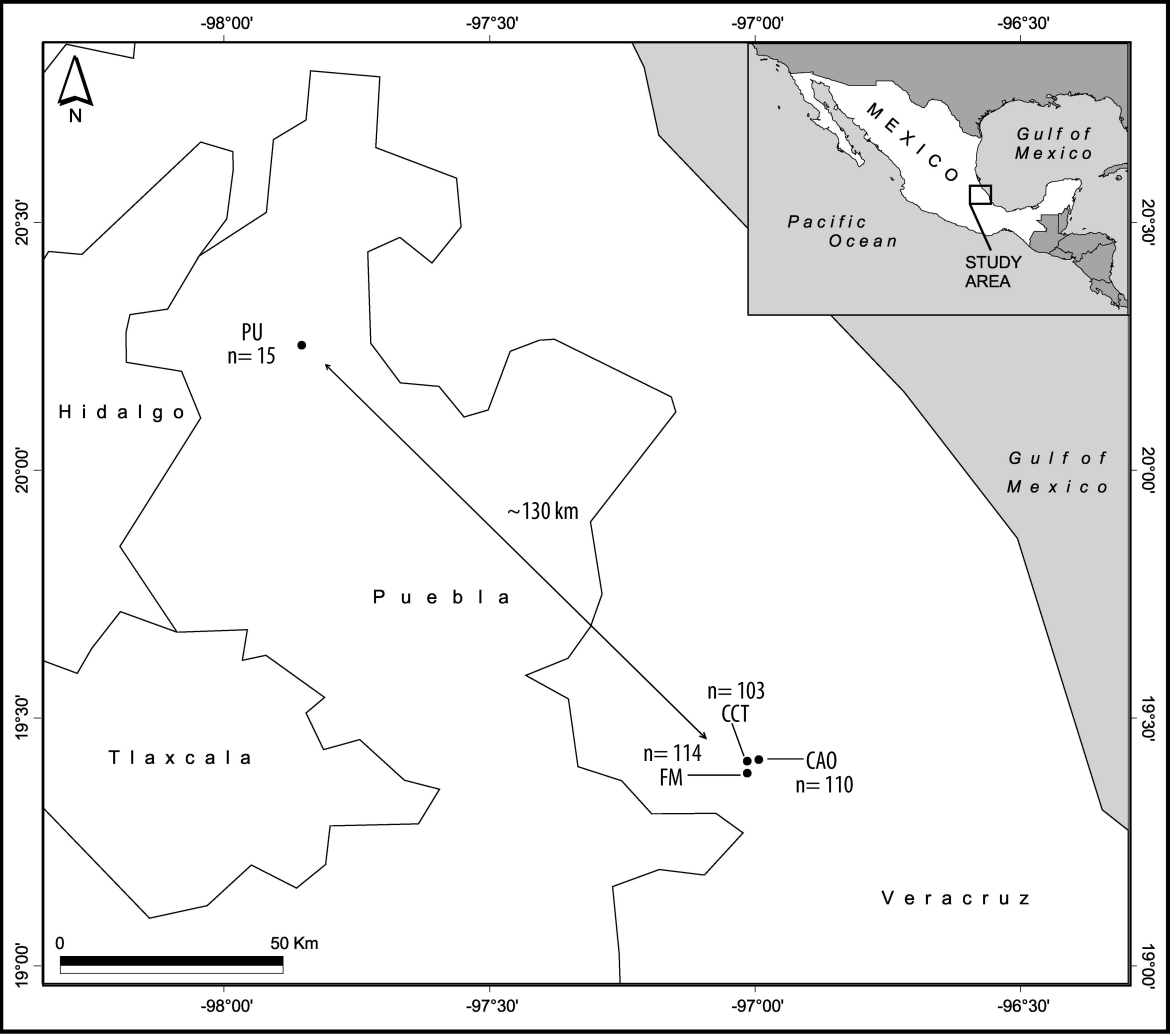
Map of localities in Veracruz and Puebla (Mexico) where samples of the *Enantia jethys* complex butterflies were collected. Localities in Veracruz: Colonia Álvaro Obregón (CAO), Camino a la Cascada Texolo (CCT), and Finca Mariposa (FM). Locality in Puebla (PU). Total number of individuals collected at each locality belonging to the three morphospecies (n).

All butterfly individuals were collected in the central region of Mexico in a total of four different localities in two states: Veracruz (Colonia Álvaro Obregón: 19°24' N, 96°58’W; Camino a la Cascada Texolo: 19°24'N, 96°59'W; Finca Mariposa: 19°23'N, 96°59'W; and Puebla: 20°15'N, 97°53'W) (Fig 1 and Table 1). All butterflies were collected in mountain cloud forests during 2015 (April—September), where the mean monthly temperature over this period varied from 23°C to 28.5°C according to locality. Butterflies were collected during consecutive days from 9:00 am to 4:00 pm using entomological nets. All individuals from the *Enantia jethys* complex were kept separately in glassine bags, labeled and geo-referenced (mobile navigation application: Locus Map). Other species of butterflies captured in the nets were released. All samples were brought back to the “Laboratorio de Genética y Evolución” (Genetics and Evolution Lab) of the “Universidad Nacional Autónoma de México” (UNAM) for their morphological identification; subsequently, abdomens from all individuals were cut, placed in absolute ethanol, and finally conserved at 4°C until DNA extraction. Voucher specimens were deposited at the “Museo de Zoología de la Facultad de Ciencias” (Science Faculty Museum of Zoology) UNAM (MZFC-UNAM) in Mexico City.

**Table 1:** Sampling information for the three species of the *Enantia jethys* complex considered in this study and collected in central Mexico.

State	Localities	*E*. *albania*					*E*. *jethys*					*E*. *mazai*				
		SC	SA ISSR	SA *COI*	SA *RpS5*	SA *Wg*	SC	SA ISSR	SA *COI*	SA *RpS5*	SA *Wg*	SC	SA ISSR	SA *COI*	SA *RpS5*	SA *Wg*
**Veracruz**	CAO	33	33	7	5	5	36	36	8	8	8	41	40	7	6	7
	CCT	15	15	7	5	7	37	37	9	8	8	51	50	6	6	5
	FM	27	27	8	8	8	50	50	11	10	9	37	36	5	4	5
**Puebla**	PU	1	1	1	1	1	0	0	0	0	0	14	14	14	14	14
**Total**		**76**	**76**	**23**	**19**	**21**	**123**	**123**	**28**	**26**	**25**	**143**	**140**	**32**	**30**	**31**

Abbreviations: Colonia Álvaro Obregón (CAO), Camino a la Cascada Texolo (CCT), Finca Mariposa (FM), number of samples collected (SC), number of samples used for analysis (SA) for inter-simple sequence repeat (ISSR), cytochrome c oxidase subunit I (*COI*), ribosomal protein subunit 5 (*RpS5*), and wingless gene (*Wg*) molecular markers.

**Fig 2:**
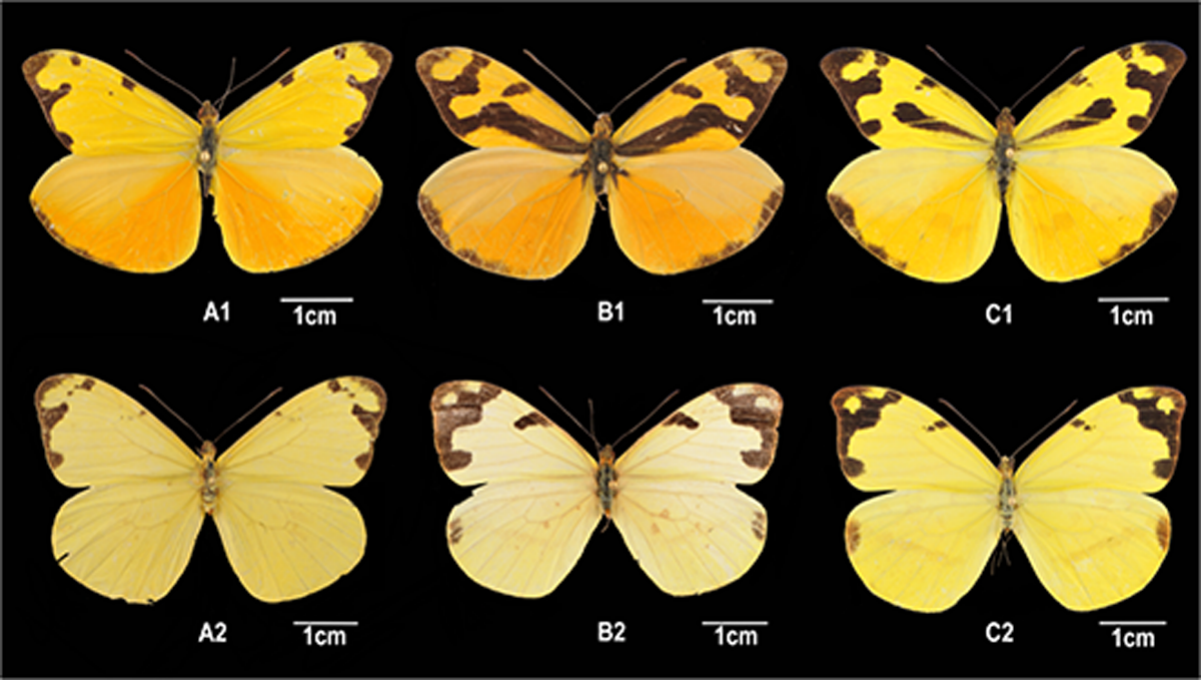
Female and male phenotypes for each morphospecies of the *Enantia jethys* complex in Mexico. *Enantia albania* (A), *Enantia jethys* (B), *Enantia mazai* (C), males (1), females (2) (photos by JM Jasso-Martínez).

Species identification (Fig 2) was based on color wing phenotypes using the descriptions from Llorente-Bousquets and colleagues [15]. Considering the difficult and sensitive identification of females (from *E*. *jethys* and *E*. *mazai*), the following steps were taken to ensure correct identification. First, we identified all males, which have wing pigmentation patterns that differ among the three species. Subsequently, females of *E*. *albania* were easily identified (presence of a diagnostic mark on the posterior wings), and separated from the remaining females. Finally, females of *E*. *jethys* and *E*. *mazai* were separated by careful observation with a stereoscopic microscope (Stemi DV4, Zeiss) and photographing each specimen. Once morphospecies (males and females) were identified, all individuals were correctly labelled and deposited (physically and in photograph) in the MZFC-UNAM collection.

### DNA extraction and genotyping

DNA was extracted from a small posterior part of the abdomen at the “Laboratorio de Ecología Molecular y Conservación" (Laboratory of Molecular Ecology and Conservation) at the “El Colegio de la Frontera Sur” research center in Chetumal, Mexico. Each DNA sample was rehydrated in ultra-pure water for one hour before extraction, which was performed using a Wizard Genomic DNA Purification Kit (Promega) following the manufacturer’s instructions. Afterwards, DNA products were stored at -20°C until amplification. Concentration of genomic DNA was determined with the Qubit®2.0 fluorometer (Invitrogen), and quality was tested using agarose gel (1% with TAE buffer 1X; Promega) and the post-gel staining method using GelRed™ (Quimica Valaner).

#### Mitochondrial DNA sequences

Mitochondrial DNA sequences were obtained at the Butterfly Diversity and Evolution Lab (Institut de Biologia Evolutiva, Barcelona, Spain). GenBank accession numbers are provided in S1 Table. They consisted of the *COI* gene that was performed on a subset of data, which consisted of 83 individuals. The target region was amplified using primers with demonstrated efficacy in butterfly studies [17, 32–33], amplifying a fragment of ~657 pb: LepR1 (5´-TAAACTTCTGGATGTCCAAAAAATCA-3’) and LepF1 (5’-ATTCAACCAATCATAAAGATATTGG-3’). The PCR mix consisted of 14.4 μl ddH_2_O, 5 μl 5X Green Buffer, 0.5 μl dNTPs mix, 0.1 μl Taq Polymerase 5 U/μl (Promega), 2 μl MgCl_2_, 2 μl of template DNA, and 0.5 μl of each primer (10 μM). Amplifications were carried out under specific conditions: initial denaturation at 92°C for 60 s, 35 cycles of denaturation at 92°C for 15 s, primer annealing temperature at 49°C for 45 s, an extension at 62°C for 150 s, and a final extension at 62°C for 7 min. Amplification products were visualized by electrophoresis using 3 μl of PCR products on a 1% agarose gel with SBYR-safe staining, at 80 V for 30 min. PCR products were sent for purification and sequencing to Macrogen Inc. (Seoul, Korea).

#### Nuclear DNA sequences

Two different nuclear genes were used: the ribosomal protein subunit 5 (*RpS5*), a relatively fast-evolving gene [34], and the wingless gene (*Wg*), involved in wing pattern formation (eyespot center formation) [35]. *RpS5* sequence was performed on a subset of data that consisted of 74 individuals. The target region was amplified using the hybrid primers with demonstrated efficacy in butterfly studies [36–37], amplifying a fragment of ~613 pb [38]: Hyb*RpS5*deg (5'-TAATACGACTCACTATAGGGATGGCNGARGARAAYTGGAAYGA-3'; forward) and Hyb*RpS5*deg (5'-ATTAACCCTCACTAAAGCGGGTTRGAYTTRGCAACACG-3'; reverse). A subset of data, consisting of 77 individuals, resulted in good sequences for *Wg* using primers with demonstrated efficacy in butterfly studies [39–40], amplifying a fragment of ~400 pb.: Lep*Wg*1 (5'-GARTGYAARTGYCAYGGYATGTCTGG-3'; forward) and Lep*Wg*2 (5'-ACTICGCARCACCARTGGAATGTRCA-3'; reverse).

The PCR mix consisted of 14.15 μl (*RpS5*) and 14.4 μl (*Wg*) ddH_2_O, 5 μl 5X Green Buffer, 0.5 μl dNTPs mix, 0.15 μl (*RpS5*) and 0.1 μl (*Wg*) Taq Polymerase 5 U/μl (Promega), 2.2 μl (*RpS5*) and 2 μl (*Wg*) of MgCl_2_, 2 μl of template DNA, and 0.5 μl of each primer (10 μM). Amplifications were carried out under specific conditions: initial denaturation at 95°C for 6 min(*RpS5*) and 3 min (*Wg*), 40 cycles of denaturation at 95°C for 30 s, primer annealing temperature at 51°C for 30 s (*RpS5*) and 60 s (*Wg*), an extension at 72°C for 90 s, and a final extension at 72°C for 10 min (*RpS5*) and 6 min (*Wg*). The protocol for visualizing the PCR products was identical to mitochondrial DNA. The PCR products were sent for purification and sequencing to Macrogen Inc. (Seoul, Korea). GenBank accession numbers are presented in S1 Table.

#### ISSR nuclear gene

We tested a total of 24 different ISSR markers. The selection of markers for this study was based on the identification of a particular band profile to facilitate the identification of each morphospecies, in addition to presenting good resolution and a high number of bands. Two ISSR markers were retained for our study: (AG)_8_Y and (GA)_8_C (Table 2).

**Table 2:** Characteristics of ISSR primers used for studying the *Enantia jethys* complex.

ISSR markers	%GC	T*m*	T*a*	*N* bands	Size (pb)
**(AG)**_**8**_**Y**	50	57.6	56	33	200–600
**(GA)**_**8**_**C**	52.9	56	54	33	200–900

Percentage of guanine and cytosine content (%GC), melting temperature (T*m*), annealing temperature (T*a*), total number of bands per primer over all localities (*N* bands), size range of the DNA fragments for each primer (size). The designation Y (C or T) was used for degenerated sites.

PCR amplifications were performed in 15 μl reaction volume containing ~20 ng of template DNA, 1.5 μl 5X Green Buffer (Promega), 200 μM dNTP (dNTP mix; Promega), 3 mM MgCl_2_ (Promega), 1 μM of primer (Integrated DNA Technologies), and 1.25 U GoTaq Flexi DNA Polymerase (Promega); finally, the volume was adjusted with ultrapure water. Amplifications were conducted in a T100 Thermal Cycler (Bio-Rad™): initial denaturation step at 94°C for 4 min, 39 cycles of denaturation at 94°C for 45 s, annealing temperature (T*a*) 54°C or 56°C depending on the ISSR primer (Table 2), extension temperature at 72°C for 2 min, and a final extension at 72°C for 10 min. DNA banding patterns were visualized by electrophoresis, performed with 3 μl of amplified products on a 2% agarose gel using 1X TAE buffer and post-staining with GelRed™ (Biotium), at 110 V for 2 h. A 100 bp DNA Ladder (Promega) was used to estimate amplification product lengths. Fragment (band) patterns were visualized and digitized using an imaging system (PhotoDoc-it, UVP®).

### DNA sequence analyses

Chromatograms of the forward and reverse sequences were edited and assembled using Geneious R9 [41], and sequences were then aligned with Geneious R9 and Mafft online [42].

Population genetic parameters were determined for each of the three morphospecies (*E*. *albania*, *E*. *jethys*, and *E*. *mazai*) for mitochondrial and nuclear genes. Program DnaSP 5.10.1 [43] was used to determine the number of haplotypes (nHap), haplotype diversity (*h*, also called genetic diversity), and nucleotide diversity (π). To determine the genetic structure among species, an analysis of molecular variance (AMOVA; 10,100 permutations) was performed using the Arlequin program 3.5 [44] under the Tamura and Nei (TrN) model, the best-fit model for nucleotide substitution, as selected by the corrected Akaike Information Criterion (AIC_C_) in jModelTest 2.1.9 [45–46]. Haplotype network analyses were performed for nuclear and mitochondrial sequences with Network software [47] though the probabilities method with the algorithm Median Joining.

For each matrix of DNA sequences, Bayesian inference was conducted in MrBayes 3.2 [48] over the Cipres web platform [49]. We used the reversible jump technique for selecting the best substitution model [50] and considered a gamma distribution with four categories and a percentage of invariant sites; the MCMCMC chains were run for 15 million generations, and the first 25% of the trees (burn-in) were discarded. A consensus tree was calculated after burn-in, and the posterior probabilities summarized in the MrBayes consensus tree were indicated on the nodes.

We performed a molecular clock analysis among species using a relaxed clock approximation with Beast 1.8.2 [51]. The analysis was performed over the web platform Cipres [49] using mean and standard deviation of the *COI* marker per Papadopoulou and colleagues [52]. We ran the analysis for 100 million generations. The ESS values were revised on Tracer software [51]. Trees sampled after *burnin* were used to construct the Maximum Clade Credibility Tree in the TreeAnotator software included in the Beast package.

### ISSR analysis

The ISSRs were treated as dominant markers; amplified fragments were scored as 1 (presence of a band represents a dominant allele) or 0 (absence represents a recessive allele). With this principle, a binary data matrix was generated for all individuals and the two ISSR markers used. Only bands that could be scored consistently among localities, and individuals that presented genetic information for all primers, were used for analysis. This can explain the low discrepancy between the numbers of collected individuals, and the number of individuals used in our analysis.

All individuals were classified in one of the three morphospecies based on their genetic ISSR profile; after which, the number of individuals of each morphospecies was compared by wing color phenotype and ISSR profile identification. For each morphospecies, we determined the parameters using GenAlex 6.5 [53–54] and Popgene 1.31 software [55], considering all localities together and individually: total number of bands (TB), number of rare bands (RB), number of private bands (PB), percentage of polymorphic loci (%*P*), and Nei’s gene diversity (*h*). Furthermore, the percentage of polymorphic loci (%*P*) and Nei’s gene diversity (*h*) were determined for “no-admixture” individuals and “admixture” individuals, considering all localities together and individually, using Popgene 1.31 software. A one-way analysis of variance (ANOVA) was used to evaluate the differences in genetic diversity (*h*) among the three morphospecies, considering all localities together and individually, among no-admixture and admixture individuals for each morphospecies, and for each morphospecies among localities, using Statistica7.0. To visualize the relationship among individuals from the three morphospecies, a principal coordinate analysis (PC_o_A) was performed in GenAlex 6.5.

The level of genetic differentiation among morphospecies was evaluated through the level of gene flow (*Nm*) and *PhiPT* parameters (*Φ*_*PT*_), an analogue to standardized *F*_*ST*_ for binary data, determined for pairs of morphospecies with 9999 permutations via AMOVA analysis. Analyses were performed using Popgene 1.31 and GenAlex 6.5 programs.

To evaluate the level of genetic homogeneity for each morphospecies, we performed a Bayesian analysis implemented in Structure v2.3.3 [56–58]. This method was designed to identify *K* (unknown) groups (or clusters), characterized by a set of allele frequencies for each locus, followed by assigning each individual, with a probability (*q*_*i*_), to one group or more, if group genotypes indicate that they are admixed. In this study, we did not look for the optimal number of populations (*K*), but we worked with a *K* = 3, which corresponds to the *a priori* three morphospecies, identified by wing phenotypes. Equally, considering the *a priori* morphospecies, we ran Structure software with the no admixture as the ancestry model, and the allele frequencies as the correlated models. The program was run 10 times for *K* = 3 to verify the homogeneity of results; for each run, the Markov chain Monte Carlo (MCMC) algorithm was run with a burn-in period of 100,000 steps, followed by 100,000 steps. Each genotype individual was assessed and allocated to one of the three clusters based on values of membership probability (*q*_*i*_). Furthermore, each individual was assigned to only one cluster if *q*_*i*_ > 0.90 (genetically “pure” individuals considered as no-admixture), otherwise they were associated with two or more clusters (admixture individuals) [19].

Finally, we performed a mean distance analysis (minimum evolution) using a heuristic search for an optimal tree, carried out with tree bisection and reconnection branch swapping. Distance analysis was performed using Paup version 4.0b10 [59] and the tree was displayed using TreeView 1.5 [60]. Negative branch lengths were allowed, but set to zero for tree-score calculation. The steepest descent options were not in effect. Starting tree(s) were obtained via neighbor joining, and bootstrap values were calculated under the same criteria.

## Results

A total of 342 butterflies in the *Enantia jethys* complex were collected and classified according to their morphological characteristics, resulting in 76 *E*. *albania* (44 females and 32 males), 123 *E*. *jethys* (53 females and 70 males), and 140 *E*. *mazai* (45 females and 95 males) (Table 1). A total of 100 randomly selected individuals were sequenced, out of which 83 provided high-quality DNA sequences for the *COI*, 75 for *RpS5*, and 77 for *Wg* genes. Furthermore, 339 samples amplified correctly with ISSR markers and were used for analysis.

### DNA sequences

A variable number of haplotypes (nHap) was observed following molecular markers, demonstrating the lowest number for *COI* (from 2 to 4) and highest for *RpS5* (14 and 26) (Table 3). The *COI* marker showed a very low variability of nHap among the three species, in contrast to the two nuclear genes. The *RpS5* gene and *Wg* gene showed the highest nHap for *E*. *jethys* and *E*. *albania*, respectively. Haplotype networks (S1 Fig) of *COI* and *RpS5* showed a clear separation among the three morphospecies, whereas for the *Wg* haplotype network, *E*. *jethys* and *E*. *mazai* share its haplotypes. Generally, haplotype diversity is high and relatively homogeneous among molecular markers and species, ranging from 0.5 to 0.9 (Table 3). Only *E*. *jethys* presented a very low value of haplotype diversity with respect to the *COI* gene. Nucleotide diversity did not show a systematic pattern that followed molecular marker or species. All values ranged from 0.002 to 0.005, with a tendency for the lowest values with the *COI* gene.

**Table 3:** Indices of haplotype diversity for the three morphospecies of the *Enantia jethys* complex based on mitochondrial (*COI*) and nuclear (*RpS5* and *Wg*) DNA sequences.

Morphospecies	N			nHap			*h*			π		
	*COI*	*RpS5*	*Wg*	*COI*	*RpS5*	*Wg*	*COI*	*RpS5*	*Wg*	*COI*	*RpS5*	*Wg*
***E*. *albania***	23	19	21	4	14	17	0.605	0.842	0.861	0.0019	0.0049	0.0052
***E*. *jethys***	28	26	25	2	26	6	0.138	0.950	0.545	0.00021	0.0050	0.0022
***E*. *mazai***	32	30	31	3	14	7	0.573	0.883	0.517	0.0032	0.0032	0.0021

Number of individuals used for analysis (N), number of haplotypes (nHap), haplotype diversity (*h*), and nucleotide diversity (π).

**Fig 3:**
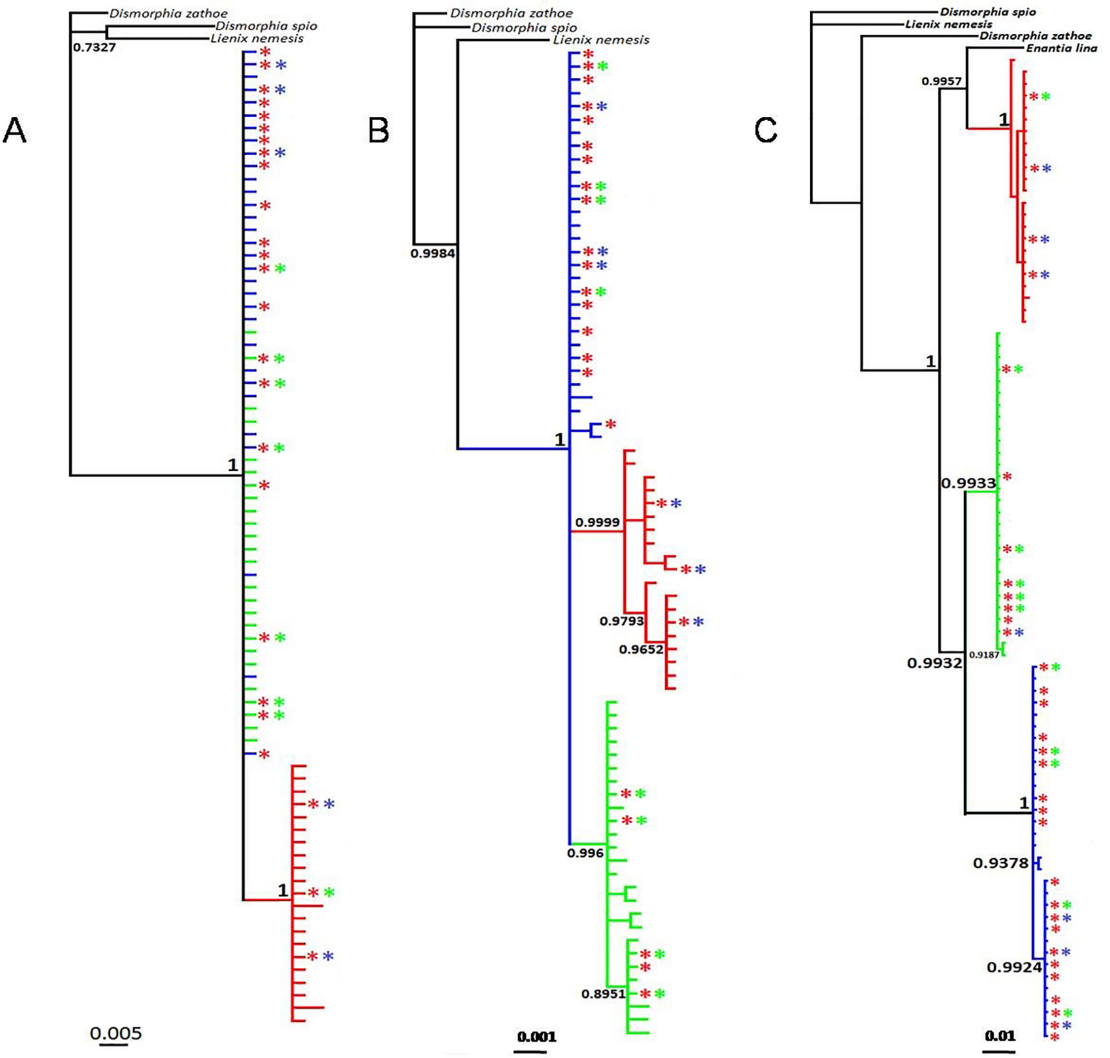
Consensus trees obtained by Bayesian inference for the *Enantia jethys* complex based on DNA sequences. (A) *Wg*, (B) *RpS5*, and (C) *COI*. Branch colors of the phylogenetic trees correspond to morphospecies identification: *E*. *albania* (red), *E*. *mazai* (blue), and *E*. *jethys* (green). Asterisks indicate the genetic profile obtained by Structure analysis based on ISSR molecular markers, where a red asterisk indicates a genetic component characteristic of *E*. *albania*, a blue asterisk indicates a genetic component of *E*. *mazai*, and a green asterisk indicates a genetic component of *E*. *jethys*. Presence of two different asterisks indicates an individual with mixed genetic components, based on Structure results.

Each molecular marker, based on sequences (*Wg*, *RpS5*, and *COI*), provided different trees (Fig 3), leading to different phylogenetic hypotheses. In the *Wg* tree (Fig 3A), two clades were observed. In the first clade, all individuals were identified as *E*. *mazai* (blue color) and *E*. *jethys* (green color) morphospecies; they were clustered with a very high posterior probability (PP) from Bayesian inference (BI). The second clade was composed only of individuals that were identified as *E*. *albania* morphospecies (red color). Both molecular markers (*RpS5* and *COI*) define three different clades corresponding to the three morphospecies, as previously reported [15–17]; however, the relationship among clades for each tree was different. The *RpS5* tree (Fig 3B) suggests that *E*. *albania* is the sister clade of *E*. *jethys*, whereas *E*. *mazai* constitutes a clade located at the base of the tree. Furthermore, the *COI* tree (Fig 3C) shows that *E*. *jethys* and *E*. *mazai* are sister groups, whereas *E*. *albania* is located at the base of the tree.

The concatenated analysis of the three molecular markers (S2 Fig) was consistent with our *COI* results (Fig 3C). There was good separation among the three species, where *E*. *albania* is a sister clade of the two other species. The molecular clock analysis (S3 Fig) identified the recent origin of the *Enantia jethys* complex species, starting around 1 Mya.

The AMOVA results varied greatly between mitochondrial and nuclear genes (S2 Table). The mitochondrial marker showed a high and significant level of differentiation among the three morphospecies, whereas both nuclear markers showed a lower but significant level of differentiation.

### ISSR

**Fig 4:**
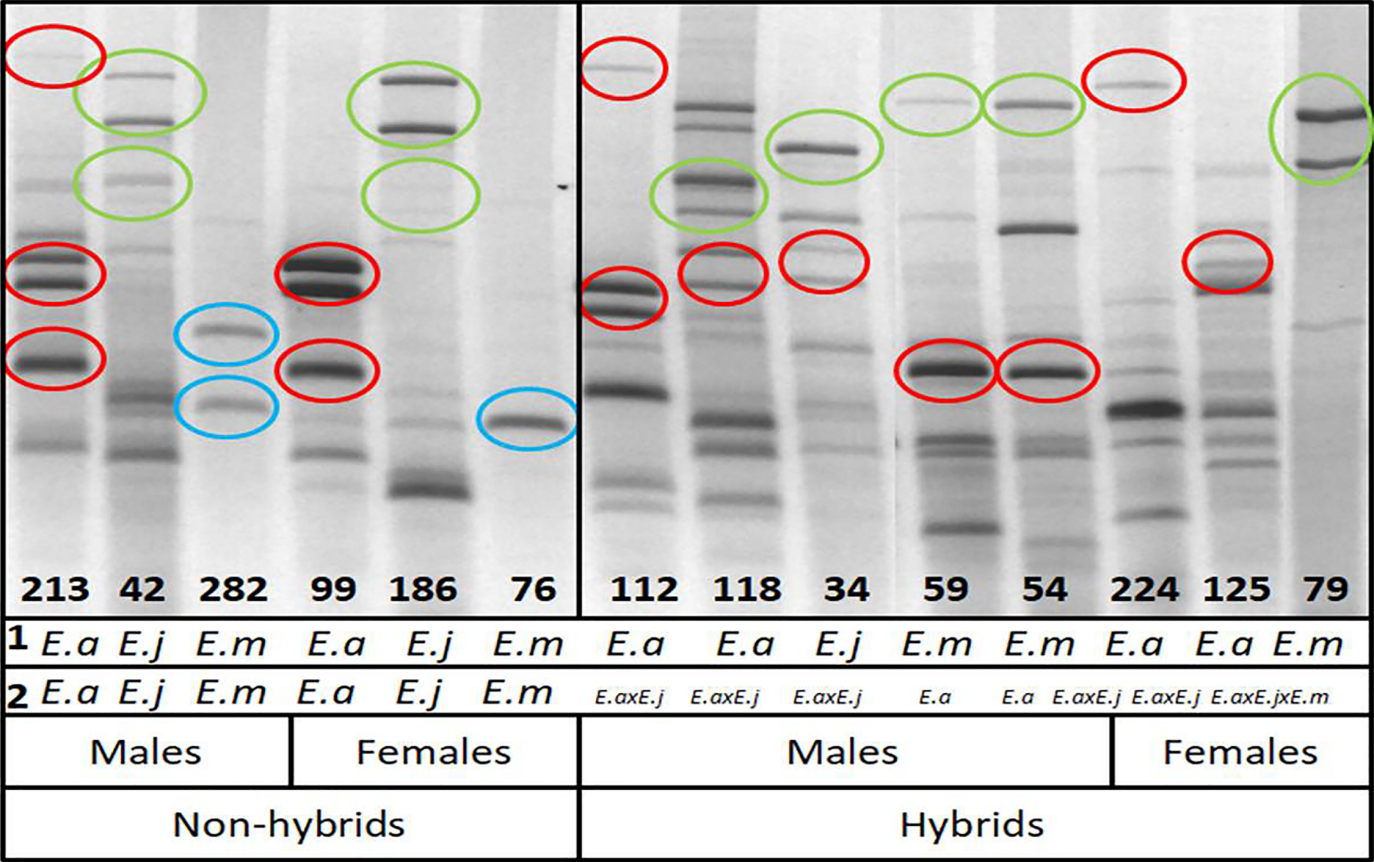
Identification of admixture and no-admixture (i.e., hybrids and non-hybrids) individuals of the *Enantia jethys* complex through ISSR profiles using (AG)_8_Y primers. *Enantia albania* (*E*. *a*), *Enantia jethys* (*E*. *j*), *Enantia mazai* (*E*. *m*). Hybrid individuals identified by Structure analysis: hybrid between *E*. *albania* and *E*. *jethys* (*E*. *a* × *E*. *j*), hybrid between *E*. *albania* and *E*. *mazai* (*E*. *a* × *E*. *m*), and hybrid among the three morphospecies (*E*. *a* × *E*. *j* × *E*. *m*). Information about morphospecies (line 1) and information about genetic profile (line 2) obtained by Structure analysis.

Of the 342 *Enantia* butterflies collected, 339 samples amplified correctly and were used for the ISSR analysis. All ISSR data are available in S3 Table. We obtained a high number of individuals for each morphospecies (76 to 140; Table 1) and a good number at each locality (Table 1), except for Puebla, where we obtained only 15 individuals belonging to two morphospecies (*E*. *albania* and *E*. *mazai*). The two ISSR molecular markers produced clear and reproducible fragments (loci) with a total of 66 ISSR fragments scored. We could not identify private bands (used to characterize species; Table 4); however, we identified some band patterns (generally two bands nearly always together; Fig 4) that allowed us to classify individuals as one of the morphospecies. The observation of ISSR band patterns enabled us to identify many individuals with mixed patterns (admixture individuals) (Fig 4).

**Table 4:** Genetic diversity in the *Enantia jethys* complex based on ISSR markers.

	*E*. *albania*			*E*. *jethys*			*E*. *mazai*		
Results for all localities									
	Total n = 76	Non-hybrids n = 63	Hybrids n = 13	Total n = 123	Non-hybrids n = 93	Hybrids n = 30	Total n = 140	Non-hybrids n = 66	Hybrids n = 74
**TB**	64	62	55	63	63	59	61	42	61
**RB**	6	5	0	3	3	4	14	16	8
**PB**	1	2	1	2	3	1	0	0	0
**%*P***	97	94	83	95	95	89	92	64	92
***h*** ± SD	0.195 ± 0.13^b^	0.193 ± 0.13	0.185 ± 0.13	0.254 ± 0.14^a^	0.263 ± 0.14	0.207 ± 0.15	0.136 ± 0.11^c^	0.071 ± 0.01	0.188 ± 0.14
		ns			*			***	
Results for each locality									
Colonia Álvaro Obregón									
	Total n = 33	Non-hybrids n = 33	Hybrids n = 0	Total n = 36	Non-hybrids n = 29	Hybrids n = 7	Total n = 40	Non-hybrids n = 17	Hybrids n = 23
**%*P***	88	88	-	93	94	65	85	45	83
***h*** ± SD	0.193 ± 0.15	0.193 ± 0.15	-	0.241 ± 0.15	0.249 ± 0.15	0.185 ± 0.17	0.141 ± 0.11	0.054 ± 0.09	0.195 ± 0.14
		na			*			***	
Camino a la Cascada Texolo									
	Total n = 15	Non-hybrids n = 10	Hybrids n = 5	Total n = 37	Non-hybrids n = 33	Hybrids n = 4	Total n = 50	Non-hybrids n = 25	Hybrids n = 25
**%*P***	88	77	70	94	92	54	88	52	86
***h*** ± SD	0.197 ± 0.14	0.186 ± 0.14	0.208 ± 0.17	0.267 ± 0.15	0.269 ± 0.15	0.176 ± 0.18	0.119 ± 0.10	0.063 ± 0.09	0.170 ± 0.13
		ns			**			***	
Finca Mariposa									
	Total n = 27	Non-hybrids n = 20	Hybrids n = 7	Total n = 50	Non-hybrids n = 31	Hybrids n = 19	Total n = 36	Non-hybrids n = 22	Hybrids n = 14
**%*P***	92	86	64	91	91	79	77	42	76
***h*** ± SD	0.182 ± 0.13	0.179 ± 0.14	0.143 ± 0.13	0.242 ± 0.15	0.258 ± 0.15	0.198 ± 0.17	0.129 ± 0.13	0.083 ± 0.13	0.181 ± 0.16
		ns			*			***	
Puebla									
	Total n = 1	Non-hybrids n = 0	Hybrids n = 1	Total n = 0	Non-hybrids n = 0	Hybrids n = 0	Total n = 14	Non-hybrids n = 2	Hybrids n = 12
**%*P***	-	-	-	-	-	-	61	17	61
***h*** ± SD	-	-	-	-	-	-	0.166 ± 0.18	0.069 ± 0.15	0.172 ± 0.18
		na			na			***	

All values are presented per morphospecies: the total number of individuals analyzed, individuals classified as non-hybrids, and those classified as hybrids. Furthermore, values are presented for all localities together and for each locality individually. Number of individuals (n); number of total bands (TB); number of rare bands (RB); number of private bands (PB); percentage of polymorphism (%*P*); Nei´s gene diversity(*h*) with standard deviation (SD); letters (a, b, c) represent intergroup differences (Tukey HSD test) for *h* among values of total individuals; probability associated with ANOVA test for *h* among no admixture and admixture categories for each morphospecies (na: not applied, ns: not significant,

* *P* < 0.05,

** *P* < 0.01,

*** *P* < 0.001).

The use of the ISSR band pattern allowed the correct identification of the majority of individuals (93.3%). Out of a total of 76 *E*. *albania* collected, 90.8% (n = 69) were confirmed by molecular ISSR technique. From the 123 *E*. *jethys* individuals, 97.6% (n = 120) were confirmed, and from the 140 *E*. *mazai* individuals, 88,6% (n = 123) were confirmed. Generally, polymorphism is very high, demonstrating values above 90% for each morphospecies (Table 4). If we consider all localities together, the morphospecies *E*. *jethys* presents a significantly higher genetic diversity than the other morphospecies, whereas *E*. *mazai* demonstrates a significantly lower genetic diversity (ANOVA test: *F*_195,2_ = 13.8, *P* < 0.001) (Table 4). For each locality, values of polymorphism and genetic diversity of each morphospecies remained similar to those observed for all localities together, even with a decrease in the sampling size. Genetic diversity was not significantly different among localities for the three morphospecies (*E*. *albania*: *F*_261,2_ = 0.19, *P* = 0.82; *E*. *jethys*: *F*_261,2_ = 0.68, *P* = 0.51; *E*. *mazai*: *F*_260,3_ = 1.49, *P* = 0.22).

**Fig 5:**
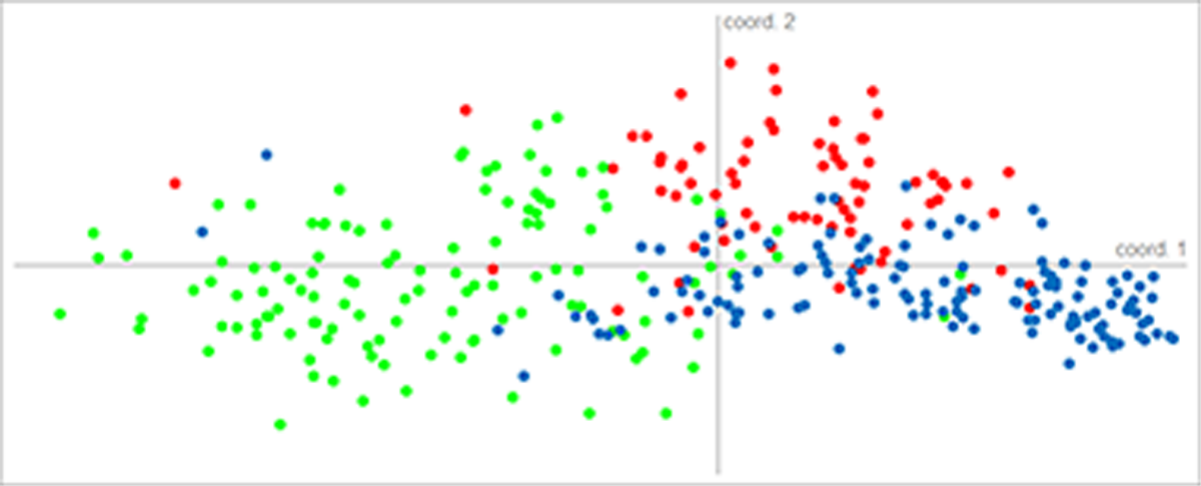
Two-dimensional scatter plot (PC_o_A) for *Enantia jethys* complex butterflies obtained through ISSR data. Colors represent each morphospecies: *E*. *albania* (red), *E*. *jethys* (green), and *E*. *mazai* (blue).

Graphical visualization of the relationship between the three morphospecies, through PC_o_A analysis, revealed good separation among morphospecies; however, there was an overlapping zone regarding individuals (Fig 5). After identification through the Structure analysis, hybrid and non-hybrid individuals were removed, and the new PC_o_A clearly separated the three morphospecies (S4 Fig).

Both genetic differentiation parameters (*Nm* and *Φ*_*PT*_) showed the same tendency: a lower level of differentiation between *E*. *albania* and the two other morphospecies (*E*. *jethys* and *E*. *mazai*), than between *E*. *jethys* and *E*. *mazai* (Table 5).

**Table 5:** Genetic differentiation among sympatric morphospecies of the *Enantia jethys* complex.

	*E*. *albania*	*E*. *jethys*	*E*. *mazai*
*E*. *albania*		11.09	14.60
*E*. *jethys*	0.116***		9.42
*E*. *mazai*	0.086***	0.142***	

Gene flow (*Nm*) above diagonal and *PhiPT* parameters (*Φ*_*PT*_) below diagonal. Probability for *Φ*_*PT*_ based on 9999 permutations:

*** *P* < 0.001.

**Fig 6:**
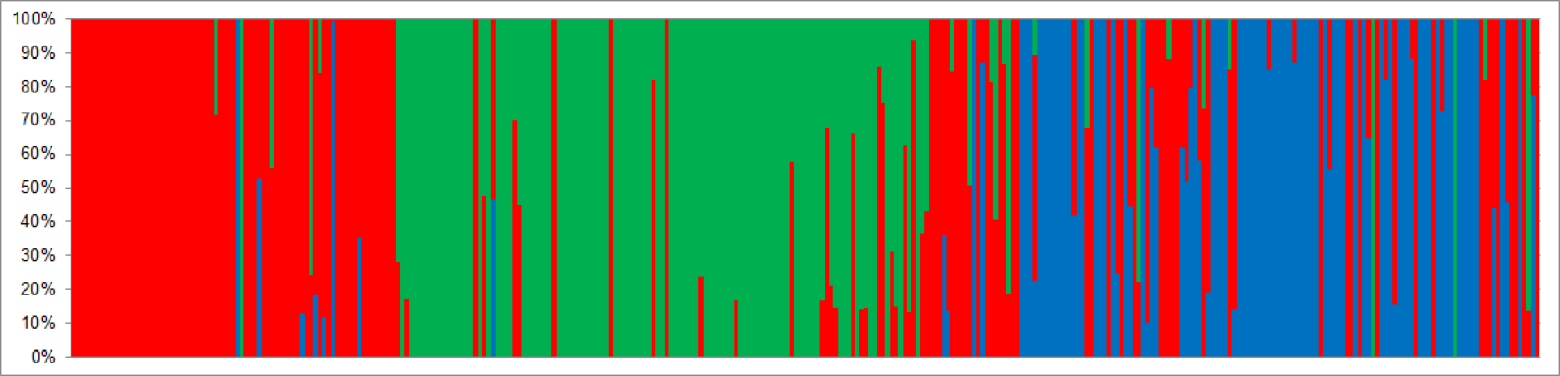
Bayesian analysis of the *Enantia jethys* complex computed by Structure software 2.3.3 with ISSR data. Results for *K* = 3 correspond to the three identified morphospecies: *E*. *albania* in red, *E*. *jethys* in green, and *E*. *mazai* in blue color. Each individual is represented by a single vertical line broken into *K* segments of length, proportional to the estimated membership probability (*q*_*i*_) in the *K* clusters.

Genetic structure results obtained with the software Structure identified individuals that represent a discrepancy between morphospecies and genetic profile (Fig 6). Based on these results, all individuals were classified as “non-hybrids” if 100% of its membership coefficient (*q*_*i*_) corresponded to the same morphospecies. They were classified as “admixture” when individuals presented a genetic profile belonging to other morphospecies, or when individuals had two or more genetic components. *Enantia albania* had the lowest level of hybrid individuals (17%), followed by *E*. *jethys* with 24%, whereas over half of *E*. *mazai* individuals (53%) were classified as hybrids. Each morphospecies presented individuals with a genetic profile belonging completely to another morphospecies, and individuals that presented a genetic profile with two, or sometimes three, different genetic components (Fig 6). Notably, a unique profile that had not been observed was the combination between the *E*. *jethys* and *E*. *mazai* profiles (blue and green together; Fig 6). Polymorphism and genetic diversity of hybrids were lower than for non-hybrids of *E*. *albania* and *E*. *jethys*; however, significant differences were only demonstrated for *E*. *jethys*. *Enantia mazai* hybrids showed a significantly higher genetic diversity than non-hybrids (Table 4). At the locality, *E*. *albania* did not present significant differences in genetic diversity between hybrids and non-hybrids. *Enantia jethys* showed a systematic, significant decrease in genetic diversity of hybrids, while *E*. *mazai* presented a systematic increase in genetic diversity of hybrid individuals (Table 4).

**Fig 7:**
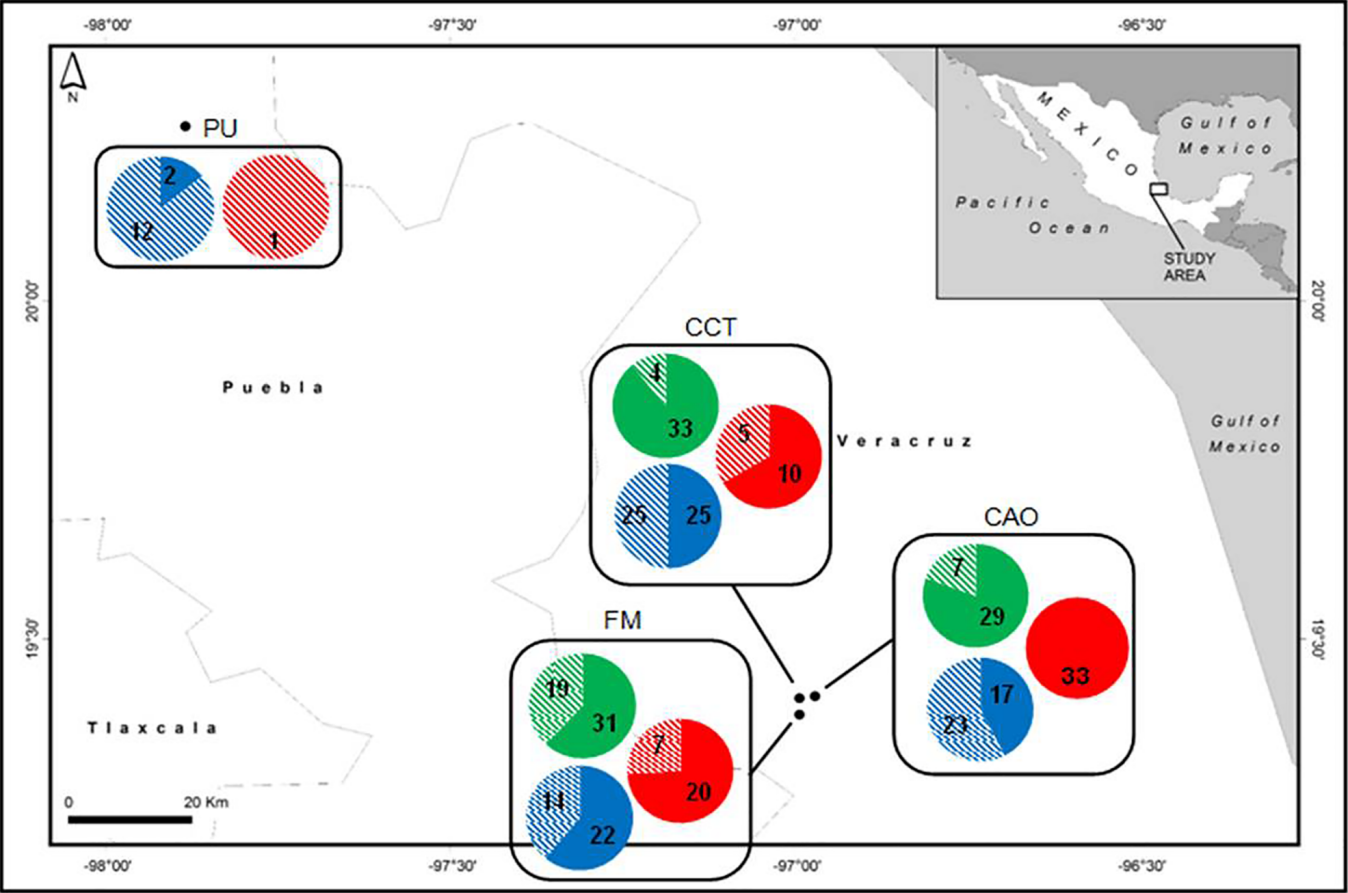
Map of the percentage of non-hybrid and hybrid individuals for the three morphospecies of the *Enantia jethys* complex in the sampled localities. *Enantia albania* (red), *E*. *mazai* (blue), and *E*. *jethys* (green). Non-hybrid individuals (full color); hybrids (hatched color); the number in each pie corresponds to the number of individuals belonging to each category.

If we consider non-hybrid and hybrid individuals from the three morphospecies at each locality (Fig 7), the three localities from Veracruz present a very similar proportion of hybrids: Colonia Álvaro Obregón 27%; Camino a la Cascada Texolo 33%; and Finca Mariposa: 35%. The Puebla locality presented a very high proportion of hybrids (87%).

From the total number of hybrid individuals (n = 117), two kinds of individuals could be distinguished: 1) individuals with only one genetic component (but different from its morphospecies), which represent 37% (n = 43); and 2) individuals with two, or three genetic components, representing 63% (n = 74). Different kinds of genetic profile combinations were found (Table 6). One individual presented a mix of the genetic profile from the three morphospecies, but interestingly no individuals combining *E*. *jethys* and *E*. *mazai* were found. Notably, almost all hybrid individuals presented an *E*. *albania* genetic component (96%, n = 112), whereas the genetic components of *E*. *jethy*s and *E*. *mazai* were found in low and similar proportions (38% and 30%, respectively).

**Table 6:** Genetic composition of the hybrids found in the *Enantia jethys* complex in Mexico based on Bayesian analysis with structure software.

Morphospecies	One genetic component			Two or three genetic components				
	*E*. *albania*	*E*. *jethys*	*E*. *mazai*	A × J	A × M	J × M	A × J × M	Total
***E*. *albania***	-	1	2	5	5	-	-	**13**
***E*. *jethys***	6	-	-	23	1	-	-	**30**
***E*. *mazai***	32	2	-	13	26	-	1	**74**
**Total**	**38**	**3**	**2**	**41**	**32**	**-**	**1**	**117**

Values represent the number of individuals for each genetic composition. To identify mix compositions: *E*. *albania* (A), *E*. *jethys* (J), and *E*. *mazai* (M).

**Fig 8:**
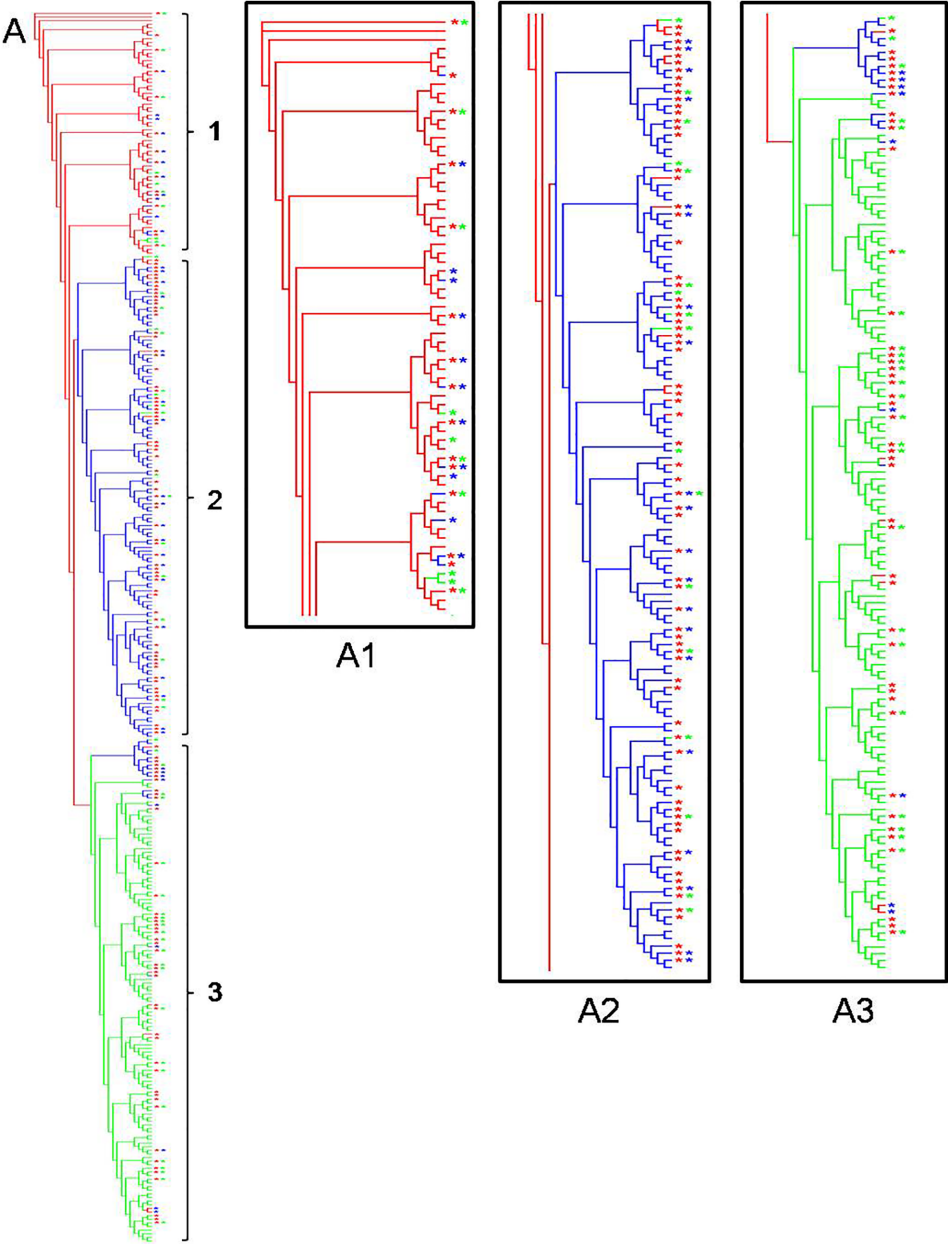
Tree obtained by distance analysis for the *Enantia jethys* complex using ISSR data. (A) Parsimony tree for the three morphospecies: *E*. *albania* (red); *E*. *mazai* (blue); and *E*. *jethys* (green). Colored asterisks correspond to the genetic profile obtained by Structure analysis. To improve the understanding of the tree, we present a zoom of each section of the tree corresponding to each morphospecies: (A1) *E*. *albania* zone of the tree (red section); (A2) *E*. *mazai* (blue section); and (A3) *E*. *jethys* (green section).

The results of the distance analysis cluster (Fig 8) showed a clear separation of the three morphospecies; however, all bootstrap values were very low. The general topology obtained by cluster analysis corresponded to the classification of individuals obtained by Structure analysis (Fig 6).

## Discussion

### Genetic diversity in the *Enantia jethys* complex

The levels of polymorphism found through ISSRs in the species of the *Enantia jethys* complex were equal or higher than those reported by other authors for butterflies [28, 30–31, 61]. Heterogeneous environments and gene exchange among populations (high connectivity) may lead to high levels of genetic variability (i.e., polymorphism) [62–63], for example *P* = 100% [26]. However, species with lower dispersal rates may attain lower values of polymorphism such as *P* ≤ 85% [29, 31]. Species of the *Enantia jethys* complex have limited flight capacity [15], which implies low dispersal rates among populations, and therefore probably cannot explain our high level of genetic diversity. Even if no formal studies on the abundance and population density of the *Enantia jethys* complex exist, we collected around 1,500 *Enantia* individuals in the field. This suggests a high population density, which could explain the high level of polymorphism.

High genetic diversity in butterflies has been associated with a wide geographic territory and the possibility of populations being isolated from others throughout evolutionary time. For example, *Papilio machaon* (Lepidoptera: Papilionidae) shows high haplotype (*h*) and nucleotide diversity (π) (*h* = 0.856, π = 0.0084) [64], as does *Aglais urticae* (Lepidoptera: Nymphalidae) (*h* = 0.9647, π = 0.00983), using mtDNA [65]. The intermediate levels of genetic diversity observed for species of the *Enantia jethys* complex could reflect their relatively small distribution ranges.

### Hybridization in the *Enantia jethys* complex

In contrast to DNA sequences, ISSR molecular markers provided evidence on the hybridization process in the *Enantia jethys* complex. Although some studies demonstrated hybridization with mitochondrial DNA sequences (*COI*) [66–67], these hybridization processes have an ancient origin. The choice of molecular markers in hybridization studies is fundamental, considering that genetic isolation is a characteristic of some regions of the genome and not of the entire genome [68]. Some markers may hybridize and/or introgress further/faster than others [68]. This can explain the differences in results between ISSR and DNA sequences. ISSR allows us to observe multilocus variation at many independent loci (random primer amplifying nuclear noncoding region), while DNA sequences show variation at a single locus (with results potentially more affected by natural selection and stochastic effects, and generally showing lower variability [69]) [70]. Consequently, ISSR molecular markers are more useful in identifying hybrids [71] and studying biological processes (e.g., diversification, dispersion, and hybridization) for species of relatively recent origin [72–74], like the hybridization processes observed in the *Enantia jethys* complex, which could be considered contemporary. Llorente-Bousquets [15] suggested that the *Enantia jethys* complex is a very recent group. Our molecular clock analysis supports this hypothesis, suggesting that the species of the *Enantia jethys* complex form a clade with a recent divergence starting around 1 Mya.

Our molecular results suggest a directional pattern for hybridization from *E*. *albania* to *E*. *jethys* and *E*. *mazai*. Some field observations (e.g., mating between species) combined with molecular information support this hypothesis. First, in the field, interspecific mating events were observed between *E*. *albania* and *E*. *jethys*, and between *E*. *mazai* and *E*. *jethys*, but no genetic profiles corresponding to a combination of *E*. *jethys* and *E*. *mazai* were identified among the 117 hybrid individuals detected in this study. Second, very few hybrids were observed in the *E*. *albania* group; and third, all hybrid individuals from the two other morphospecies contained a genetic component of *E*. *albania*. These results could suggest some reproductive barriers among species of the complex and differences in the viability of hybrids, with the existence of a prezygotic barrier (occuring after mating or gametic contact, thereby reducing the probability of fertilization [75]) between *E*. *mazai* and *E*. *jethys*, while mating involving *E*. *albania* does not appear to have a strong prezygotic barrier and hybrids are probably fertile. Prezygotic isolation is favored by natural selection as a process against unfit hybrids, as shown for *Agrodiaetus* butterflies [76]. Many studies on a variety of taxa ([75] for review) have shown that prezygotic barriers are important barriers to prevent gene flow between closely related species. Other types of reproductive barriers are reported in other butterflies, such as *Aricia* and *Polyommatus*, two non-sister cryptic butterfly species for which barriers seem to be precopulatory [77], or between two sympatric sister species (*Leptidea sinapis* and *L*. *reali*) that present premating reproductive isolation [78].

Many cases of interspecific hybridization have been reported for Lepidoptera (non-exhaustive list: [66–67, 79]). It is not surprising to observe hybridization in the *Enantia jethys* complex, considering that closely related species are more likely to hybridize [80], and that hybridization appears to be facilitated by wider sympatric distributions of parental species [81]. Furthermore, species of the *Enantia jethys* complex are subject to the same environmental pressures as they share the same oviposition and larval host plants [15], which may also facilitate opportunities for interspecific mating.

A study on Lepidoptera showed that hybrid individuals may present intermediate morphology [82]. Hybrid individuals of the *Enantia jethys* complex did not show intermediate morphology, however *E*. *mazai* males have a wide variation of morphotypes (S5 Fig). The hybridization process in natural populations may act in different ways, such as increasing genetic variation and new gene combinations, which favor novel adaptations [12, 24], or reducing hybrid fitness [13] because of a postzygotic barrier [75]. Our study demonstrates an interesting case with different effects of hybridization on genetic diversity. First, *E*. *albania* hybrids showed a lower, although not significant, genetic diversity value (*h*) than non-hybrids, probably a reflection of sample size. Second, hybrid individuals of *E*. *jethys* presented a low but significant decrease in genetic diversity value (*h*), which could reflect a reduction in hybrid fitness. Finally, hybrid individuals of *E*. *mazai* showed an important and significant increase in genetic diversity value (*h*). This is not surprising because non-hybrid *E*. *mazai* individuals presented the lowest value of genetic diversity. Therefore, when they cross with individuals with higher genetic diversity, hybrids presented higher genetic diversity values (*h*), which could result in novel adaptations. Nevertheless, additional ecological, behavioral, chromosomal, and genomic studies will be necessary to test our hypothesis.

### Phylogenetic relationships in the *Enantia jethys* complex

A previous study of the *Enantia jethys* complex using morphological data supported the existence of three distinct species: *E*. *albania*, *E*. *jethys*, and *E*. *mazai*, subsequently subdivided in subspecies *E*. *mazai mazai* and *E*. *m*. *diazi* [15]. The first study that investigated this species complex through DNA sequences (*COI* barcode) [17] validated the existence of the species *E*. *albania*, *E*. *jethys*, and *E*. *mazai*, without any evidence for subspecies or deep intraspecific differentiation. However, these authors discovered a sister clade of *E*. *mazai* composed of specimens that were morphologically indistinguishable from *E*. *albania*, suggesting the existence of a potential cryptic species originating in a specific geographic area (Ahuaxentitla, Puebla, Mexico). Using a multilocus approach (mitochondrial and nuclear markers), we confirmed the existence of three clades in the *Enantia jethys* complex in agreement with the results reported by Jasso-Matínez and colleagues [17]. The different topology recovered through the *Wg* marker (*E*. *mazai* and *E*. *jethys* in the same clade and shared haplotypes, Panel A in S1 Fig) could be explained by incomplete lineage sorting, as observed in other Lepidoptera studies [83–84]. Incomplete lineage sorting frequently results in different patterns between nuclear and mitochondrial markers [85], which is common in species of recent divergence [84]. To understand this phenomenon, a multiple independent loci analysis was used to generate a robust phylogenetic hypothesis (S2 Fig), as recommended by Edwards and Bensch [86]. Overall, our results confirmed the existence of three well-defined groups, corresponding to the three different morphospecies previously described [15–17].

## Supporting information

S1 Fig Haplotype networks from DNA sequences. A) *Wg*, B) *RpS5*, and C) *COI*. Black circles are the mutational steps.
(TIF)

S2 Fig Bayesian concatenated tree. *COI*, *RpS5*, and *Wg* for the *Enantia jethys* complex. *E*. *albania* (red), *E*. *jethys* (green), and *E*. *mazai* (blue).
(TIF)

S3 Fig Molecular clock analysis for the *Enantia jethys* complex. *E*. *albania* (red), *E*. *jethys* (green), and *E*. *mazai* (blue).
(TIF)

S4 Fig Principal coordinate analysis (PC_o_A). Obtained after removing hybrid individuals from the dataset. *E*. *albania* (red), *E*. *jethys* (green), and *E*. *mazai* (blue).
(TIF)

S5 Fig Illustration of the wide range of phenotypes for *Enantia mazai* male individuals. Pictures are organized according to a gradient of brown wing pigmentation, from lower to higher extent of brown spots. A) Phenotype very similar to a male of *E*. *albania*; B, C, and D) Common phenotypes; E) Phenotype very similar to *E*. *jethys* males.
(TIF)

S1 Table GenBank accession numbers for *COI*, *RpS5*, and *Wg* genetic markers. (DOC)

S2 Table Genetic differentiation for the *Enantia jethys* complex. Evaluated by analysis of molecular variance using mitochondrial (*COI*) and nuclear (*RpS5* and *Wg*) DNA sequences.
(DOC)

S3 Table ISSR data. (XLS)
