# Supplementary figures and images for: Molecular evidence of hybridization in sympatric populations of the Enantia jethys complex (Lepidoptera: Pieridae)
Source: PLoS One. 2018 May 17;13(5):e0197116. doi: 10.1371/journal.pone.0197116 (PMC5957354; doi:10.1371/journal.pone.0197116)

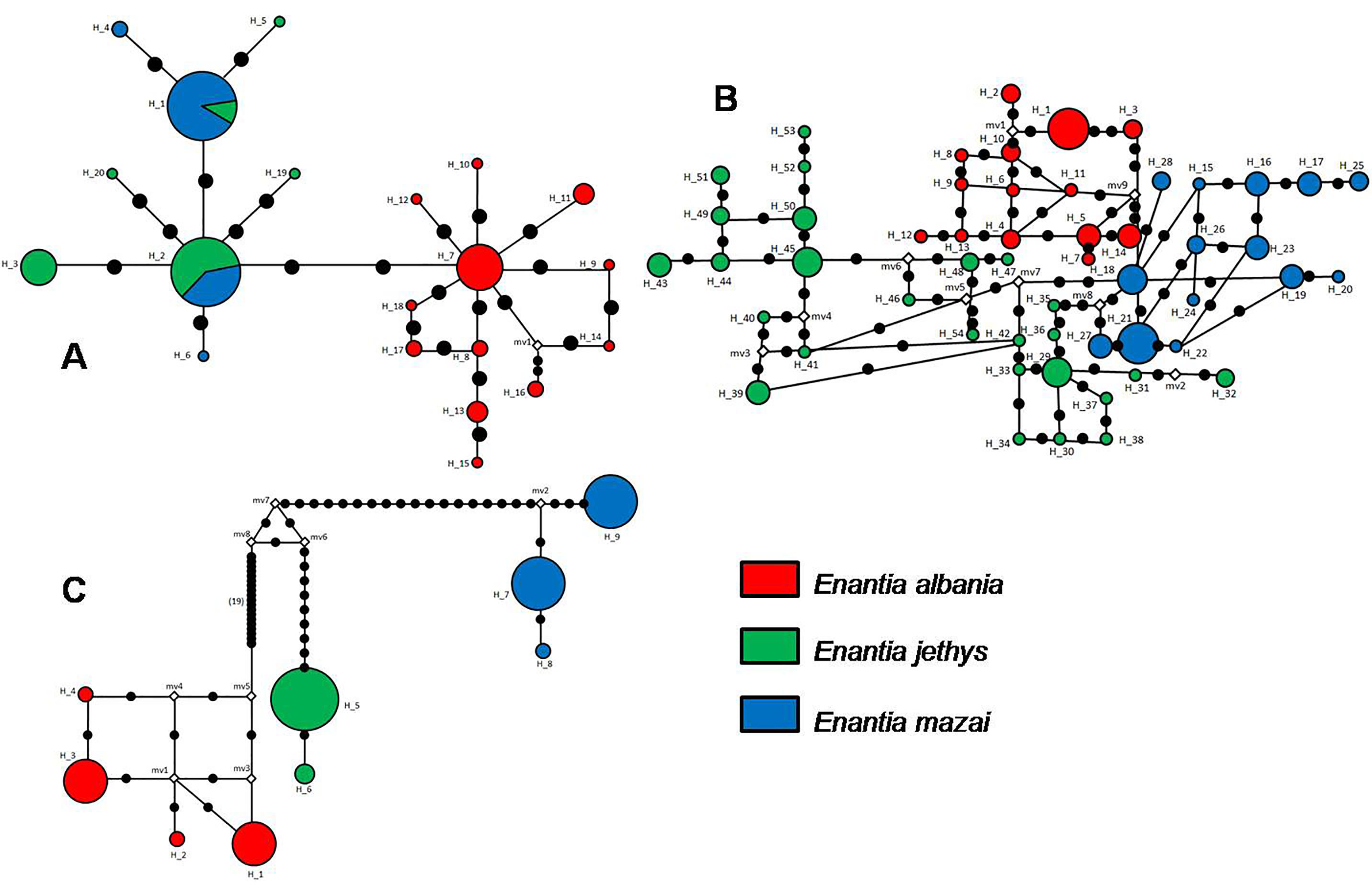

Supplement: S1 Fig — A) Wg, B) RpS5, and C) COI. Black circles are the mutational steps. (TIF) [file pone.0197116.s001.tif]

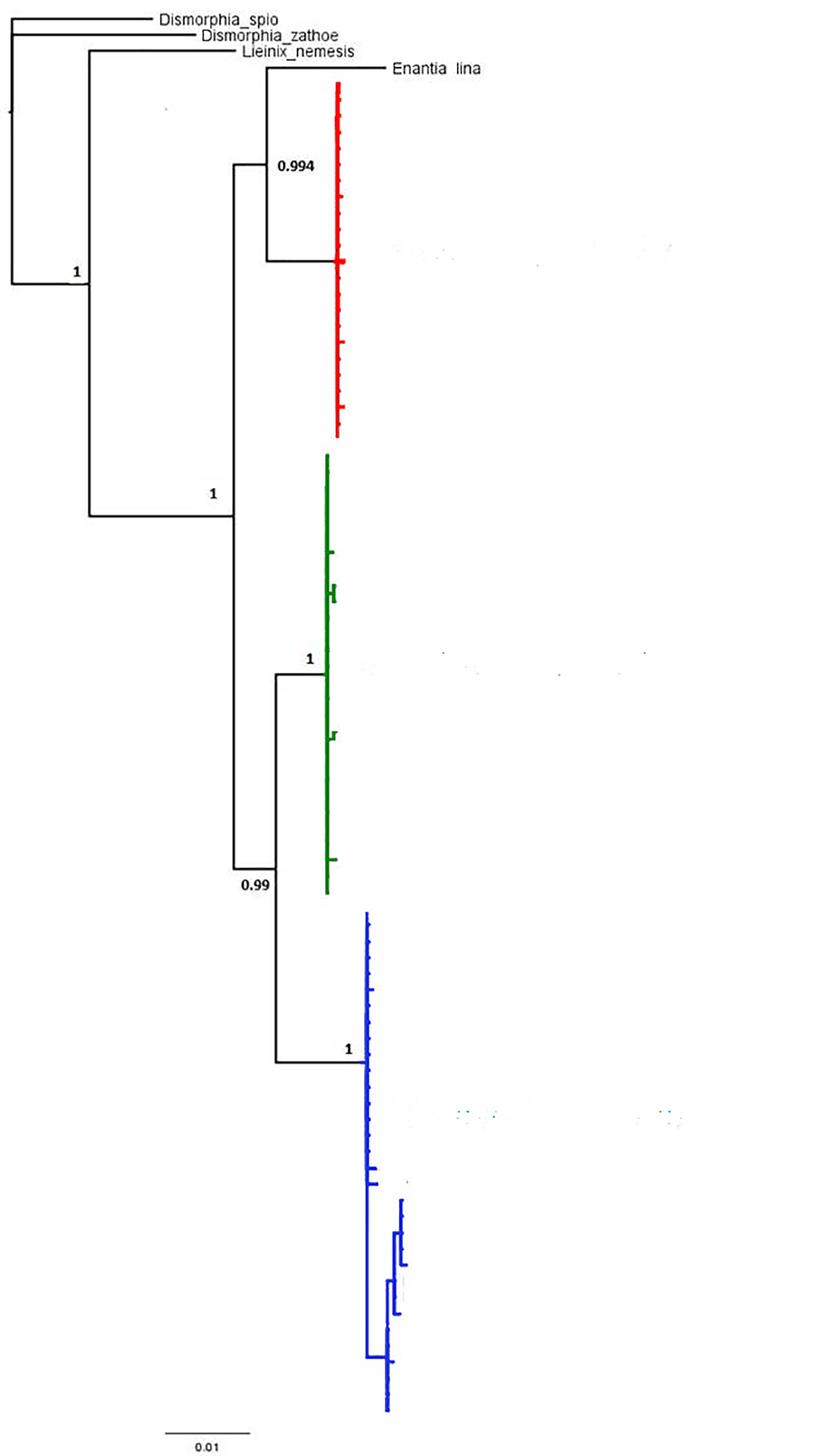

Supplement: S2 Fig — COI, RpS5, and Wg for the Enantia jethys complex. E. albania (red), E. jethys (green), and E. mazai (blue). (TIF) [file pone.0197116.s002.tif]

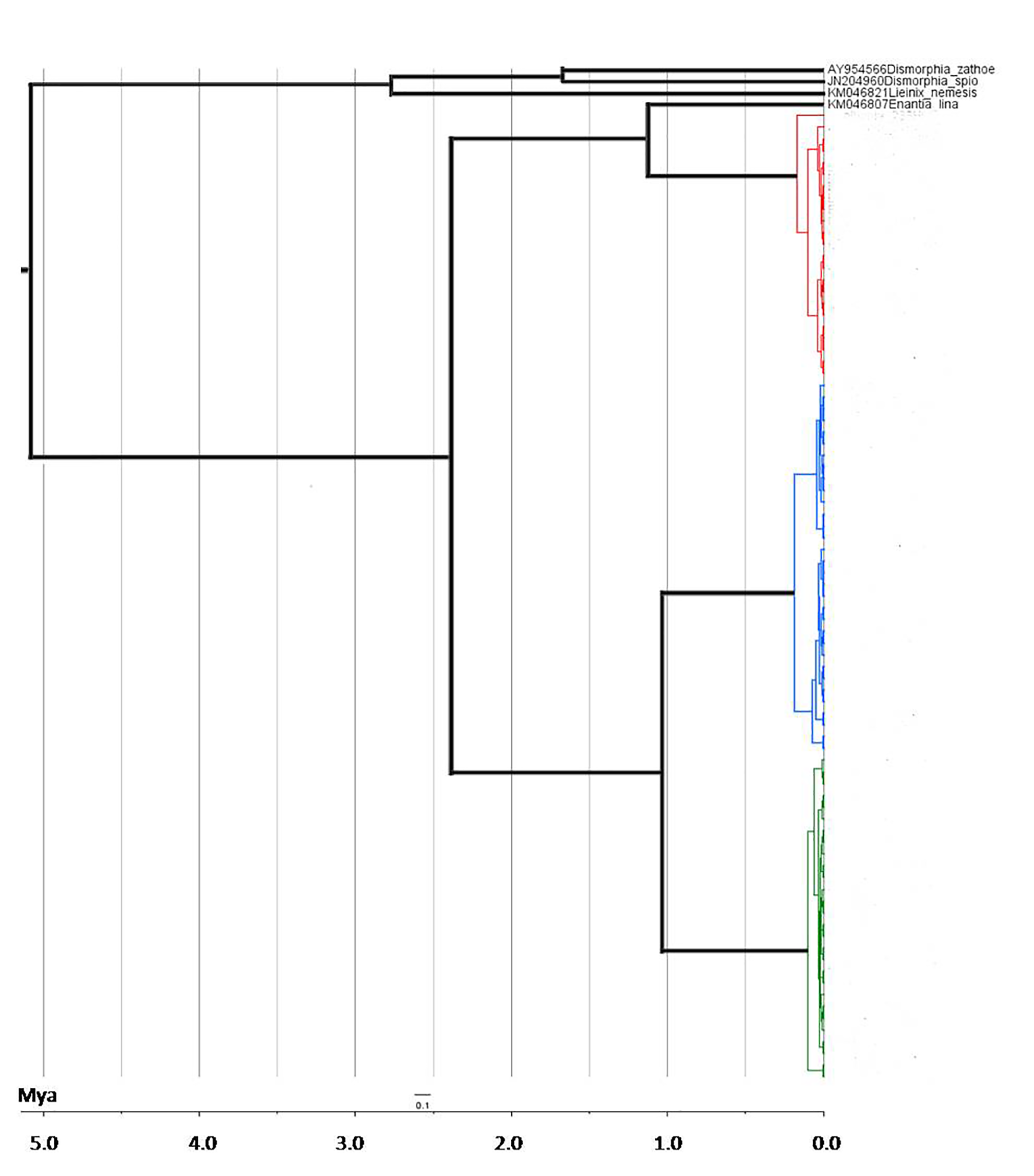

Supplement: S3 Fig — E. albania (red), E. jethys (green), and E. mazai (blue). (TIF) [file pone.0197116.s003.tif]

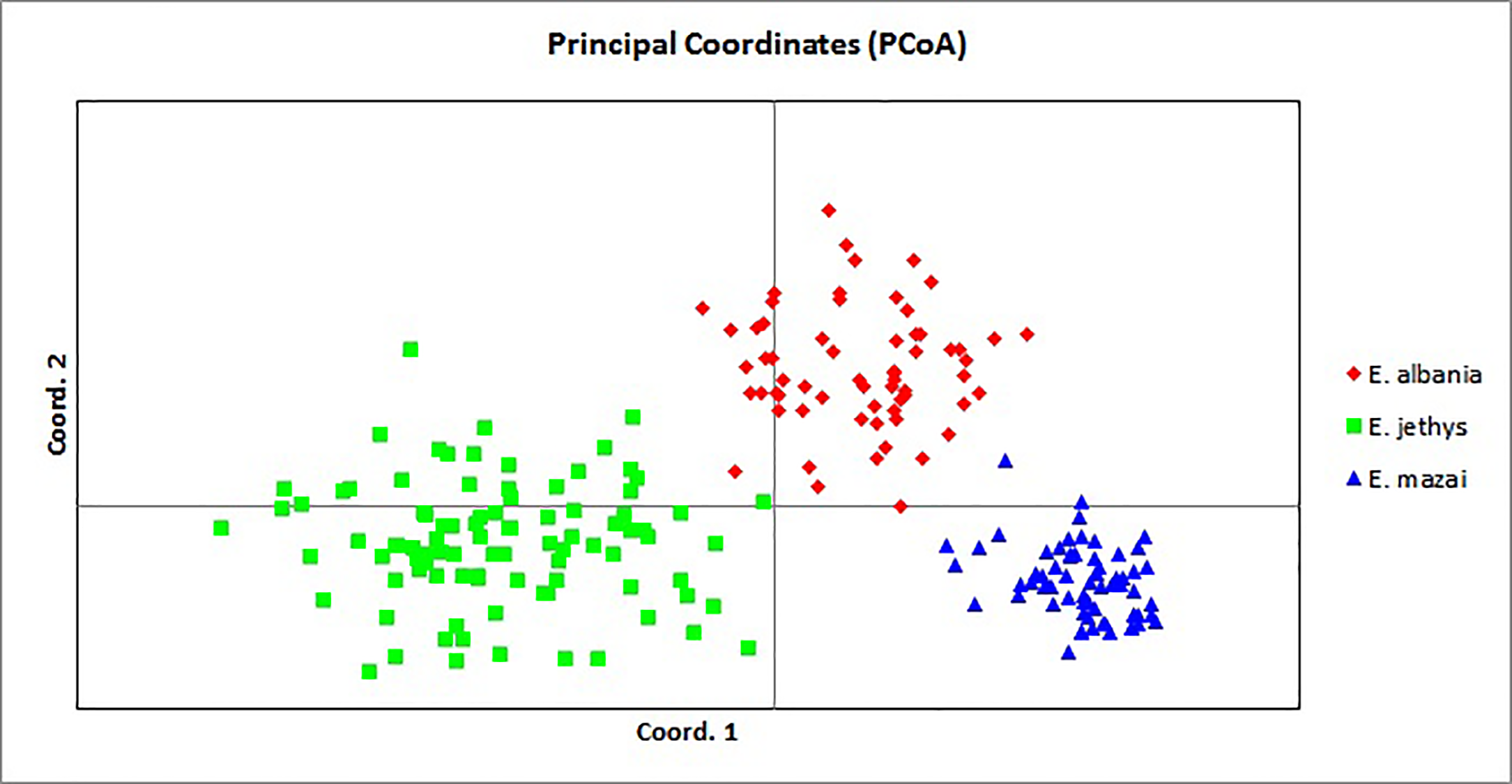

Supplement: S4 Fig — Obtained after removing hybrid individuals from the dataset. E. albania (red), E. jethys (green), and E. mazai (blue). (TIF) [file pone.0197116.s004.tif]

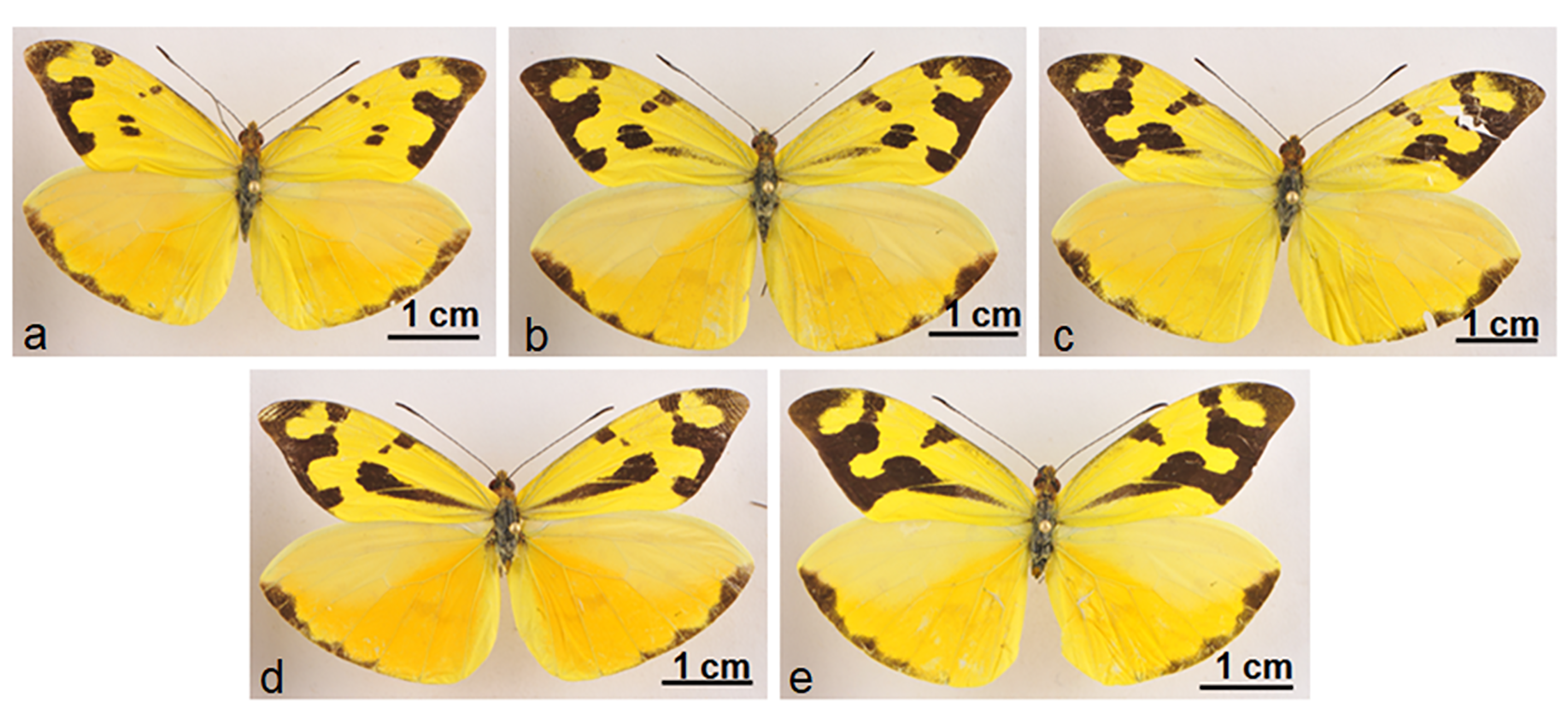

Supplement: S5 Fig — Pictures are organized according to a gradient of brown wing pigmentation, from lower to higher extent of brown spots. A) Phenotype very similar to a male of E. albania; B, C, and D) Common phenotypes; E) Phenotype very similar to E. jethys males. (TIF) [file pone.0197116.s005.tif]
