# Supplementary material for: Molecular evidence of hybridization in sympatric populations of the Enantia jethys complex (Lepidoptera: Pieridae)
Source: PLoS One. 2018 May 17;13(5):e0197116. doi: 10.1371/journal.pone.0197116 (PMC5957354; doi:10.1371/journal.pone.0197116)
Supplement: S1 Table — (DOC) [file pone.0197116.s006.doc]

|  |  |  | **GenBank acession numbers** | | |
| --- | --- | --- | --- | --- | --- |
| **Label** | **Species** | **Location** | ***COI*** | ***RpS5*** | ***Wg*** |
| E_albania_2MCAO | *Enantia albania* | Colonia Álvaro Obregón (CAO), Veracruz, Mexico | - | - | MH137178 |
| E_albania_4MCAO | *Enantia albania* | Colonia Álvaro Obregón (CAO), Veracruz, Mexico | MH143400 | MH137049 | - |
| E_albania_8MCAO | *Enantia albania* | Colonia Álvaro Obregón (CAO), Veracruz, Mexico | MH143401 | MH137051 | - |
| E_albania_112MCCT | *Enantia albania* | Camino a la Cascada Texolo, Veracruz, Mexico | MH143402 | - | MH137180 |
| E_albania_115MCCT | *Enantia albania* | Camino a la Cascada Texolo, Veracruz, Mexico | MH143403 | MH137044 | MH137176 |
| E_albania_116MCCT | *Enantia albania* | Camino a la Cascada Texolo, Veracruz, Mexico | MH143404 | - | MH137186 |
| E_albania_121HCCT | *Enantia albania* | Camino a la Cascada Texolo, Veracruz, Mexico | MH143405 | MH137045 | MH137187 |
| E_albania_213MCCT | *Enantia albania* | Camino a la Cascada Texolo, Veracruz, Mexico | MH143406 | MH137061 | MH137184 |
| E_albania_216MFM | *Enantia albania* | Finca Mariposa, Veracruz, Mexico | MH143407 | MH137059 | MH137175 |
| E_albania_220MFM | *Enantia albania* | Finca Mariposa, Veracruz, Mexico | MH143408 | MH137062 | MH137185 |
| E_albania_221MFM | *Enantia albania* | Finca Mariposa, Veracruz, Mexico | MH143409 | MH137053 | MH137172 |
| E_albania_230HFM | *Enantia albania* | Finca Mariposa, Veracruz, Mexico | MH143410 | MH137055 | MH137183 |
| E_albania_238HFM | *Enantia albania* | Finca Mariposa, Veracruz, Mexico | MH143411 | MH137056 | MH137182 |
| E_albania_13MCAO | *Enantia albania* | Colonia Álvaro Obregón (CAO), Veracruz, Mexico | MH143412 | MH137052 | - |
| E_albania_26HCAO | *Enantia albania* | Colonia Álvaro Obregón (CAO), Veracruz, Mexico | MH143413 | MH137060 | MH137171 |
| E_albania_99HCAO | *Enantia albania* | Colonia Álvaro Obregón (CAO), Veracruz, Mexico | MH143414 | MH137058 | MH137170 |
| E_albania_122HCCT | *Enantia albania* | Camino a la Cascada Texolo, Veracruz, Mexico | MH143415 | MH137046 | MH137173 |
| E_albania_123HCCT | *Enantia albania* | Camino a la Cascada Texolo, Veracruz, Mexico | MH143416 | MH137050 | MH137174 |
| E_albania_218MFM | *Enantia albania* | Finca Mariposa, Veracruz, Mexico | MH143417 | MH137047 | MH137188 |
| E_albania_225HFM | *Enantia albania* | Finca Mariposa, Veracruz, Mexico | MH143418 | MH137054 | MH137192 |
| E_albania_239HFM | *Enantia albania* | Finca Mariposa, Veracruz, Mexico | MH143419 | MH137048 | MH137189 |
| E_albania_31HCAO | *Enantia albania* | Colonia Álvaro Obregón (CAO), Veracruz, Mexico | MH143420 | - | MH137177 |
| E_albania_22HCAO | *Enantia albania* | Colonia Álvaro Obregón (CAO), Veracruz, Mexico | MH143421 | - | MH137179 |
| E_albania_342HZA2 | *Enantia albania* | Puebla, Mexico | MH143399 | MH137057 | MH137181 |
| E_jethys_37MCAO | *Enantia jethys* | Colonia Álvaro Obregón (CAO), Veracruz, Mexico | MH143449 | MH137108 | MH137147 |
| E_jethys_42MCAO | *Enantia jethys* | Colonia Álvaro Obregón (CAO), Veracruz, Mexico | MH143448 | MH137113 | MH137159 |
| E_jethys_49MCAO | *Enantia jethys* | Colonia Álvaro Obregón (CAO), Veracruz, Mexico | MH143447 | MH137114 | MH137164 |
| E_jethys_52MCAO | *Enantia jethys* | Colonia Álvaro Obregón (CAO), Veracruz, Mexico | MH143446 | MH137110 | MH137143 |
| E_jethys_80HCAO | *Enantia jethys* | Colonia Álvaro Obregón (CAO), Veracruz, Mexico | MH143445 | MH137095 | MH137167 |
| E_jethys_89HCAO | *Enantia jethys* | Colonia Álvaro Obregón (CAO), Veracruz, Mexico | MH143444 | MH137102 | MH137152 |
| E_jethys_97HCAO | *Enantia jethys* | Colonia Álvaro Obregón (CAO), Veracruz, Mexico | MH143443 | MH137115 | MH137151 |
| E_jethys_159MCCT | *Enantia jethys* | Camino a la Cascada Texolo, Veracruz, Mexico | MH143442 | MH137092 | MH137148 |
| E_jethys_164MCCT | *Enantia jethys* | Camino a la Cascada Texolo, Veracruz, Mexico | MH143422 | MH137100 | MH137155 |
| E_jethys_174MCCT | *Enantia jethys* | Camino a la Cascada Texolo, Veracruz, Mexico | MH143441 | MH137105 | MH137141 |
| E_jethys_180HCCT | *Enantia jethys* | Camino a la Cascada Texolo, Veracruz, Mexico | MH143440 | MH137106 | MH137162 |
| E_jethys_189HCCT | *Enantia jethys* | Camino a la Cascada Texolo, Veracruz, Mexico | MH143439 | MH137111 | MH137157 |
| E_jethys_192HCCT | *Enantia jethys* | Camino a la Cascada Texolo, Veracruz, Mexico | MH143438 | - | - |
| E_jethys_202HCCT | *Enantia jethys* | Camino a la Cascada Texolo, Veracruz, Mexico | MH143437 | MH137118 | MH137194 |
| E_jethys_208HCCT | *Enantia jethys* | Camino a la Cascada Texolo, Veracruz, Mexico | MH143436 | MH137099 | MH137163 |
| E_jethys_243MFM | *Enantia jethys* | Finca Mariposa, Veracruz, Mexico | MH143435 | MH137103 | MH137166 |
| E_jethys_250MFM | *Enantia jethys* | Finca Mariposa, Veracruz, Mexico | MH143434 | MH137101 | MH137193 |
| E_jethys_256MFM | *Enantia jethys* | Finca Mariposa, Veracruz, Mexico | MH143433 | MH137109 | MH137158 |
| E_jethys_297HFM | *Enantia jethys* | Finca Mariposa, Veracruz, Mexico | MH143432 | MH137112 | - |
| E_jethys_299HFM | *Enantia jethys* | Finca Mariposa, Veracruz, Mexico | MH143431 | MH137097 | MH137169 |
| E_jethys_306HFM | *Enantia jethys* | Finca Mariposa, Veracruz, Mexico | MH143430 | MH137107 | MH137154 |
| E_jethys_313HFM | *Enantia jethys* | Finca Mariposa, Veracruz, Mexico | MH143429 | - | - |
| E_jethys_321HFM | *Enantia jethys* | Finca Mariposa, Veracruz, Mexico | MH143428 | MH137116 | MH137190 |
| E_jethys_327MFM | *Enantia jethys* | Finca Mariposa, Veracruz, Mexico | MH143427 | MH137093 | MH137165 |
| E_jethys_328HFM | *Enantia jethys* | Finca Mariposa, Veracruz, Mexico | MH143426 | MH137094 | MH137153 |
| E_jethys_73HCAO | *Enantia jethys* | Colonia Álvaro Obregón (CAO), Veracruz, Mexico | MH143425 | MH137098 | MH137161 |
| E_jethys_169MCCT | *Enantia jethys* | Camino a la Cascada Texolo, Veracruz, Mexico | MH143424 | MH137104 | MH137156 |
| E_jethys_317HFM | *Enantia jethys* | Finca Mariposa, Veracruz, Mexico | MH143423 | MH137096 | MH137191 |
| E_mazai_57MCAO | *Enantia mazai* | Colonia Álvaro Obregón (CAO), Veracruz, Mexico | MH143462 | MH137073 | MH137150 |
| E_mazai_62MCAO | *Enantia mazai* | Colonia Álvaro Obregón (CAO), Veracruz, Mexico | MH143461 | MH137069 | MH137142 |
| E_mazai_68MCAO | *Enantia mazai* | Colonia Álvaro Obregón (CAO), Veracruz, Mexico | MH143460 | MH137087 | MH137134 |
| E_mazai_72MCAO | *Enantia mazai* | Colonia Álvaro Obregón (CAO), Veracruz, Mexico | MH143459 | - | MH137139 |
| E_mazai_76HCAO | *Enantia mazai* | Colonia Álvaro Obregón (CAO), Veracruz, Mexico | MH143458 | MH137088 | MH137149 |
| E_mazai_84HCAO | *Enantia mazai* | Colonia Álvaro Obregón (CAO), Veracruz, Mexico | MH143457 | MH137090 | MH137129 |
| E_mazai_142MCCT | *Enantia mazai* | Camino a la Cascada Texolo, Veracruz, Mexico | MH143456 | MH137063 | MH137195 |
| E_mazai_148MCCT | *Enantia mazai* | Camino a la Cascada Texolo, Veracruz, Mexico | MH143455 | MH137074 | MH137168 |
| E_mazai_154MCCT | *Enantia mazai* | Camino a la Cascada Texolo, Veracruz, Mexico | MH143467 | MH137064 | MH137136 |
| E_mazai_185HCCT | *Enantia mazai* | Camino a la Cascada Texolo, Veracruz, Mexico | MH143468 | MH137080 | MH137132 |
| E_mazai_195HCCT | *Enantia mazai* | Camino a la Cascada Texolo, Veracruz, Mexico | MH143469 | MH137075 | MH137138 |
| E_mazai_214HCCT | *Enantia mazai* | Camino a la Cascada Texolo, Veracruz, Mexico | MH143470 | MH137068 | - |
| E_mazai_277MFM | *Enantia mazai* | Finca Mariposa, Veracruz, Mexico | MH143471 | - | MH137135 |
| E_mazai_286MFM | *Enantia mazai* | Finca Mariposa, Veracruz, Mexico | MH143472 | MH137083 | MH137144 |
| E_mazai_293MFM | *Enantia mazai* | Finca Mariposa, Veracruz, Mexico | MH143473 | MH137076 | MH137160 |
| E_mazai_303HFM | *Enantia mazai* | Finca Mariposa, Veracruz, Mexico | MH143474 | MH137117 | MH137137 |
| E_mazai_94HCAO | *Enantia mazai* | Colonia Álvaro Obregón (CAO), Veracruz, Mexico | MH143475 | MH137091 | MH137140 |
| E_mazai_269MFM | *Enantia mazai* | Finca Mariposa, Veracruz, Mexico | MH143476 | MH137066 | MH137133 |
| E_mazai_329MAA | *Enantia mazai* | Puebla, Mexico | MH143477 | MH137071 | MH137125 |
| E_mazai_330MUZ | *Enantia mazai* | Puebla, Mexico | MH143478 | MH137072 | MH137146 |
| E_mazai_331MZA1 | *Enantia mazai* | Puebla, Mexico | MH143479 | MH137065 | MH137131 |
| E_mazai_332HZA1 | *Enantia mazai* | Puebla, Mexico | MH143480 | MH137079 | MH137127 |
| E_mazai_333MZA1 | *Enantia mazai* | Puebla, Mexico | MH143481 | MH137070 | MH137128 |
| E_mazai_334MZA1 | *Enantia mazai* | Puebla, Mexico | MH143451 | MH137077 | MH137130 |
| E_mazai_335HZA1 | *Enantia mazai* | Puebla, Mexico | MH143452 | MH137067 | MH137120 |
| E_mazai_336MZA1 | *Enantia mazai* | Puebla, Mexico | MH143453 | MH137089 | MH137119 |
| E_mazai_337MZA2 | *Enantia mazai* | Puebla, Mexico | MH143454 | MH137084 | MH137123 |
| E_mazai_338HZA2 | *Enantia mazai* | Puebla, Mexico | MH143463 | MH137085 | MH137121 |
| E_mazai_339MZA2 | *Enantia mazai* | Puebla, Mexico | MH143464 | MH137086 | MH137124 |
| E_mazai_340MZA2 | *Enantia mazai* | Puebla, Mexico | MH143465 | MH137081 | MH137145 |
| E_mazai_341HZA2 | *Enantia mazai* | Puebla, Mexico | MH143466 | MH137078 | MH137122 |
| E_mazai_343HZA2 | *Enantia mazai* | Puebla, Mexico | MH143450 | MH137082 | MH137126 |
