# Supplementary material for: Molecular evidence of hybridization in sympatric populations of the Enantia jethys complex (Lepidoptera: Pieridae)
Source: PLoS One. 2018 May 17;13(5):e0197116. doi: 10.1371/journal.pone.0197116 (PMC5957354; doi:10.1371/journal.pone.0197116)
Supplement: S2 Table — Evaluated by analysis of molecular variance using mitochondrial (COI) and nuclear (RpS5 and Wg) DNA sequences. (DOC) [file pone.0197116.s007.doc]

| **Source of variation** | **df** | **Sum of squares** | **Variance components** | **% of variation** |
| --- | --- | --- | --- | --- |
| ***COI*** |  |  |  |  |
| **Among species** | 2 | 931.784 | 16.96759 | 96.5 |
| **Within species** | 80 | 48.977 | 0.61221 | 3.5 |
| ***RpS5*** |  |  |  |  |
| **Among species** | 2 | 306.537 | 3.09035 | 70.3 |
| **Within species** | 147 | 191.971 | 1.30592 | 29.7 |
| ***Wg*** |  |  |  |  |
| **Among species** | 2 | 141.574 | 1.36963 | 67.6 |
| **Within species** | 153 | 100.193 | 0.65486 | 32.4 |
